# Remote‐sensing Based Assessment of Long‐term Riparian Vegetation Health in Proximity to Agricultural Lands with Herbicide Use History

**DOI:** 10.1002/ieam.4144

**Published:** 2019-06-22

**Authors:** Foad Yousef, Mekonnen Gebremichael, Lula Ghebremichael, Jeffrey Perine

**Affiliations:** ^1^ Department of Civil and Environmental Engineering, University of California Los Angeles, Los Angeles California USA; ^2^ Syngenta Crop Protection Greensboro North Carolina USA

**Keywords:** Riparian vegetation, Landsat, NDVI, Herbicide, Remote sensing

## Abstract

Riparian ecosystems provide various ecosystem services including habitat for a variety of plant and animal communities, biofiltering, and stabilizing stream and river systems. Due to their location, riparian zones often share long borders with agricultural fields where herbicides are commonly applied to eliminate unwanted plants. There is a general concern that exposure of riparian vegetation to off‐target drifted herbicides may adversely impact their health and diversity. We utilized the Normalized Difference Vegetation Index (NDVI) to investigate the long‐term (between 1992 and 2011) trend of riparian vegetation health at 17 locations in the Midwest and Great Plains areas of the United States, where herbicide usage was likely most intense. Assessment of NDVI data demonstrated that long‐term vegetation health did not decline for the studied riparian zones located in proximity to croplands during spring months (April and May). During summer (June and July), while the long‐term vegetation health did not decline for the majority of the sites, there were a few cases in Kansas and Nebraska with a decline in vegetation health (negative‐trending NDVI). Cluster analysis of the negative‐trending NDVI pixels showed that the majority of these pixels were randomly distributed throughout these riparian sites, indicating a lack of shared common causing factors. Similarly, proximity analysis suggested that distance from croplands was not associated with the decline of vegetation health found in these sites, suggesting that exposure to herbicide drift may not be a plausible factor because this would have shown higher impact on pixels closer to the cropland. Changes in canopy coverage and vegetation diversity also did not show any dependence on distance from croplands. Finally, the remote‐sensing–based NDVI data sets used provide only an indirect way of assessing the impact of herbicide drift, and therefore, further work based on field survey data is recommended to completely isolate the impacts of herbicides. *Integr Environ Assess Manag* 2019;15:528–543. © 2019 The Authors. *Integrated Environmental Assessment and Management* published by Wiley Periodicals, Inc. on behalf of Society of Environmental Toxicology & Chemistry (SETAC)

## Key Points

- We assessed the riparian vegetation health (VH) of the United States Great Plains for 2 decades.
- Spring VH remained unchanged or increased at all 17 sites.
- Summer VH declined at few sites in Kansas and Nebraska.
- We found no association between decline in VH and distance to croplands.

## 1 INTRODUCTION

A riparian zone is the land area that occurs along aquatic systems (stream or river, lakes, and reservoirs) and is located between the low and high watermark (Gregory et al. 1991; Martin et al. 1999) where it hosts a variety of vegetation and animal species. Thus, riparian zones play a key role in defining the ecological functioning of terrestrial and aquatic environments (Niman et al. 1993). Riparian ecosystems also define and shape many physical (water clarity, temperature, erosion, etc.) and hydrological (flood, etc.) characteristics of the land and water in their proximity (Bowler et al. 2012). These systems are often in vicinity to urban and agricultural environments and, therefore, have been subject to various perturbations since the European settlement (Obedzinski et al. 2001). Major disturbing factors to riparian ecosystems are urbanization, invasive species (Howe and Knopf 1991; Fierke and Kauffman 2006), mining (Andrews et al. 1985), recreation (Johnson and Carothers 1982), and agriculture (Nilsson and Berggren 2000; Poff et al. 2011). Although some of these disruptions have been documented (Nilsson and Berggren 2000), little is known about the long‐term vegetation health of riparian zones located in proximity to agricultural fields with a history of herbicides usage.

In the Midwest and Great Plains region of the United States, riparian environments often share long borders with various croplands (e.g., corn, soybean) and due to this proximity, there is a concern that off‐field herbicide spray drift could culminate in exposure to plant communities making up the riparian habitat (Dalton et al. 2015). Many of these herbicides are often nonselective (Hartley and Kidd 1983) and, therefore, can potentially harm off‐target vegetation outside the farm area. Repeated applications of herbicides raise concern about their potential for adverse impacts on riparian ecosystems by potentially decreasing the general health of vegetation and eliminating the more vulnerable species. The most vulnerable riparian zones to drifted herbicides are expected to be located proximal to agricultural fields in regions with history of long‐term herbicide application and high wind speed.

The objective of the present study is to assess the trend (if any) in the long‐term vegetation health and diversity of riparian ecosystems in the vicinity of agricultural areas of Midwest and Great Plains regions where there has been historically repeated and intensive use of herbicides. We employed temporal trend analysis based on the long‐term Normalized Difference Vegetation Index (NDVI) data sets derived from Landsat satellite imageries in order to detect any changes in the long‐term vegetation health of riparian zones. We hypothesized that any negative trend in the vegetation health would indicate increases in unfavorable growing conditions (exposure to herbicides, water stress, etc.) over a long time period. The NDVI is a commonly used index to monitor ecosystem health (e.g., Prakash et al. 2017; Bento et al. 2018). In addition, spatial proximity tests were performed to examine the role of distance (between the riparian zone and the cropland) on vegetation health, crop canopy, and diversity of riparian ecosystems. We hypothesized that riparian vegetation located downwind and near croplands (i.e., herbicide application area) would have the greatest potential long‐term impact from wind‐displaced herbicides compared to those located farthest from the cropland.

## 2 DATA AND METHODOLOGY

Our methodology involved 1) selection of riparian zones potentially vulnerable to herbicide exposures; 2) use of 20 y (1992–2011) of NDVI data, derived from Landsat satellite imageries, to assess the trend in greenness (proxy for vegetation health); 3) use of canopy coverage (CC) and vegetation diversity (VD) data derived from Landsat imageries; 4) temporal trend analysis of NDVI at different spatial scales and distances from cropland; 5) changes in CC between 2 different years (2001 and 2011) as a function of distance from cropland; and 6) variation in VD as a function of distance from cropland.

### 2.1 Riparian zone selection

#### 2.1.1 Riparian zone mapping

**Figure 1:**
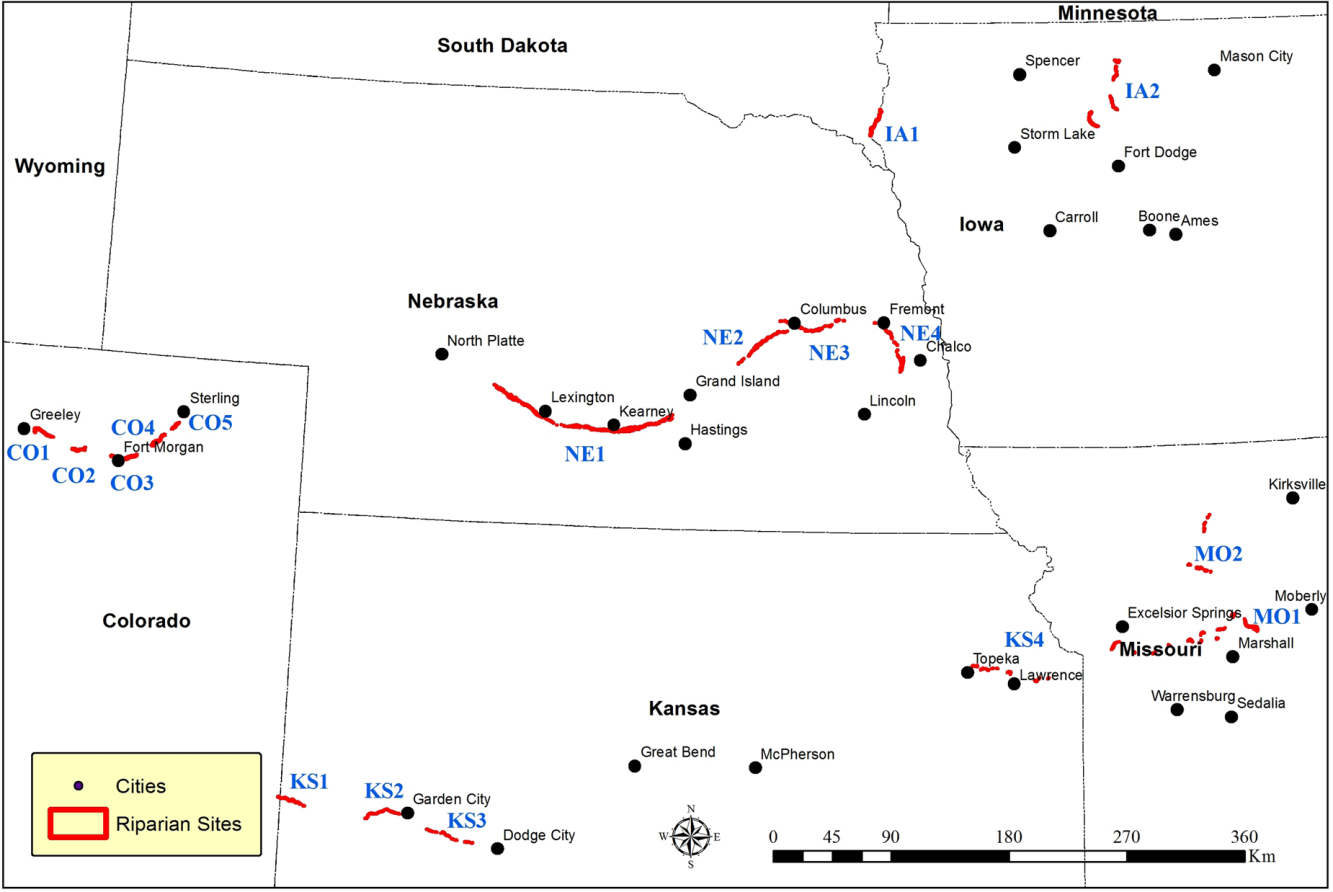
Selected study sites which had both “high‐wind” and “heavy‐herbicides use” history. Labels in all caps denote site names. Site names are abbreviated state names followed by a number (e.g., CO1 = Colorado site 1, etc.).

The boundary of a riparian environment is defined by the interplay of multiple hydrological, topographic, and biological factors. Delineation of riparian sites is currently an active area of research, with few publicly available riparian maps. In the present study, riparian zone boundaries (Figure 1) were determined using preexisting maps and by creating new maps following traditional on‐screen mapping methods. For the states of Nebraska and Colorado, the United States Fish and Wildlife Services riparian maps were used (2017; https://www.fws.gov/wetlands/Data/State-Downloads.html#Riparian). For those states where riparian maps were not available (Indiana, Missouri, and Kansas), riparian zone delineation was performed following the method by Johnson and Zelt (2005).

Selection of vulnerable riparian sites was based on 3 criteria: herbicide usage, wind speed (average speed for April and May greater than 9 miles per hour [mph]), and wind crossing agricultural fields in the direction of the riparian zone (wind direction).

#### 2.1.2 Herbicide usage

The US Geological Service (USGS) provides the most publicly available comprehensive series of herbicide usage maps for the entire country. The USGS National Water‐Quality Assessment (NAWQA) program (https://water.usgs.gov/nawqa/pnsp/usage/maps/compound_listing.php) has annual agricultural herbicide‐use maps for 480 herbicides and other pesticides for the period 1992 to 2014 (Baker and Stone 2015). A long‐term average distribution map was constructed for each of the top 10 herbicides (glyphosate, atrazine, metam‐sodium, S‐metolachlor, acetochlor, dichloropropene, 2,4‐D methyl bromide, chloropicrin, and pendimethalin). These top ten represent about 90% of overall herbicide usage for 2006 and 2007 (Grube et al. 2011). We employed an additive method to merge the geographical extent of each of these herbicides and produce a comprehensive herbicide coverage map following the method by Thelin and Stone (2013).

#### 2.1.3 Wind speed

Wind is a critical factor in the physical transport of herbicides away from farmlands. Wind speed and the spray droplet size distribution are the most important factors that determine how far herbicides travel before depositing on a nontarget surface. Farmlands located in regions with consistently high wind speed could pose greater risks of herbicide drift into riparian zones. We obtained wind speed data at 30‐m height from the National Renewable Energy Laboratory (NREL, http://www.nrel.gov/; Draxl et al. 2015), and converted it to surface wind speed using a power law equation relating wind speed to height (Spera and Richards 1979). We considered regions with average annual wind speeds greater than 9 mph as “high‐wind” areas. Commercial herbicide product labels commonly restrict wind speeds during application to 10 or 15 mph, so this represents a high‐end range for potential downwind exposures.

#### 2.1.4 Wind direction

Nontarget areas, including riparian zones, are potentially exposed to airborne spray drift only when located downwind from herbicide application. To identify vulnerable riparian areas, we sought locations where the wind direction was most consistently from farmland to the riparian zone. Wind direction data were obtained from the Automated Surface Observing System (ASOS 1998) stations (https://mesonet.agron.iastate.edu/).

The intersection of the 3 criteria listed above defined the location and boundary of the most vulnerable riparian sites. Because uninterrupted high wind speeds are a common feature of the Great Plains and many parts of Midwest region (Klink 1999), wind direction and location of riparian zones with respect to herbicide use area became mainly the driving factors in the selection of riparian sites.

### 2.2 Remote sensing data

Vegetation functioning (health, diversity, distribution, etc.) has been routinely assessed via satellite imagery (Fensholt et al. 2006; Van Leeuwen et al. 2013; Mazzarino and Finn 2016). Due to the narrow geometry of riparian ecosystems, high‐resolution satellite imagery (such as Landsat) is appropriate for such regions. In the present study, we used the following data products obtained from Landsat imageries: NDVI to assess vegetation health, and CC and VD to assess any changes in diversity of plant species.

#### 2.2.1 Data acquisition

We used the Landsat 4 and 5 archive to reduce the variations among satellites and to capture the longest available time frame (1992–2011). The Landsat visible and infrared bands have a 30 m × 30 m pixel size and the satellite revisits each location on earth every 16 d. The NDVI data were directly downloaded from the Earth Resources Observation and Science (EROS) Science Processing Architecture (ESPA, https://espa.cr.usgs.gov/, Landsat 4‐5 image courtesy of US Geological Survey). Pixels contaminated with ice, snow, and clouds were removed using the pixel quality band (pixel_qa band) provided by ESPA. Tree CC data were downloaded from the National Land Cover Database (NLCD, http://www.mrlc.gov/index.php, National Land Cover Database courtesy of US Geological Survey). Gap Analysis Program (GAP, http://gapanalysis.usgs.gov/, Gap Analysis Program Database courtesy of US Geological Survey) was used for detailed vegetation type distribution and change analysis.

#### 2.2.2 Normalized difference vegetation index

The NDVI quantifies vegetation greenness by measuring the normalized difference between the reflectance in the near‐infrared and red region. The unique feature of chlorophyll in plants is such that it has a very low reflectivity in the red region and a very high reflectivity in the near‐infrared region, resulting in a very high NDVI value. Thus, higher NDVI values are indicative of greener and healthier vegetation. On the other hand, lower NDVI values may indicate weaker photosynthetic activity and overall poorer vegetation health, due to likely unfavorable growing conditions (e.g., water stress, exposure to herbicide). The NDVI is typically used as indicator to monitor ecosystem health (e.g., Bento et al. 2018; Flores‐Cardenas et al. 2018; Mariano et al. 2018) and crop phenology (e.g., Mashaba et al. 2016; Martin and Latheef 2017; Inurreta‐Aguirre et al. 2018). A number of studies (e.g., Thelen et al. 2004; Dicke et al. 2012; Lewis et al. 2014; Prakash et al. 2017) have demonstrated that NDVI is an effective index in detecting herbicide damage on plants.

Therefore, in the present study, NDVI was used as an indicator for the vegetation health of riparian ecosystems. Using archived Landsat 4‐5 imagery courtesy of US Geological Survey, NDVI values were extracted from the scenes available between April and July for years from 1992 to 2011. If 2 images were available for the same month, the average was used to produce the monthly value. For seasonal analysis, images from the months of April and May were stacked to construct the spring data set, and images from June and July were stacked to construct the summer season data set. As the final step before data analysis, all of the areas outside of the riparian sites were masked out.

#### 2.2.3 Canopy coverage

Canopy coverage data represent the percent tree canopy in each pixel and, therefore, can indirectly quantify the density of hardwood for a given area (Homer et al. 2007; Coulston et al. 2012). Canopy coverage data typically taken during peak growing seasons are publicly available only for 2 y: 2001 and 2011. Because these data were derived from Landsat imageries, they have preserved the 30 m × 30 m pixel size. We performed change detection analysis by taking the difference in CC between the 2 observation years. The resulting map had both negative and positive pixel values, representing lost and gained CC between 2001 and 2011, respectively.

#### 2.2.4 Vegetation diversity, Gap Analysis Project

The GAP data set offers enhanced land cover types for the contiguous United States. These data sets are also derived from Landsat imagery and have the same spatial resolution (30 m × 30 m pixels). The GAP complements the National Vegetation Classification System (NVC) structure by providing the ecological system data set. Ecological system data set uses the NatureServe (2017) Ecological System Classification (Comer et al. 2003) to map vegetation classes. In our study, the extent and variety of vegetation classes were considered as indicative of VD in the riparian sites. The GAP data set is available only for year 2001. Nonvegetation land‐cover pixels (e.g., urban, agricultural) were removed before further analysis. Finally, the number of available ecological vegetation types was determined for each riparian site.

## 3 STATISTICAL ANALYSIS

We performed various statistical analyses on each data set, as shown in Supplemental Data Figure S1. A brief description of the statistical methods applied is presented here.

### 3.1 Trend analysis

The purpose of the trend analysis is to detect any systematic long‐term change in the NDVI, a proxy for vegetation health. We performed linear temporal trend analysis by fitting a linear model to the NDVI time series data. Significance testing (*F*‐test) was performed on the slope estimate of the linear model to test significance of the linearity (i.e., trend) and direction. The null hypothesis is that the slope is 0, indicating no trend. The trend analysis has 3 possible outcomes: no change in NDVI (slope not significantly different from 0), positive trend in NDVI (positive slope significantly different from 0), or negative trend in NDVI (negative slope significantly different from 0). The trend analysis was done at monthly and seasonal timescales. The seasonal data were obtained by averaging the monthly NDVI values within each season (Spring: April and May; Summer: June and July). We performed the trend analysis at 2 spatial scales: riparian‐site (areal‐) average, and pixel (30 m × 30 m).

#### 3.1.1 Site‐average temporal trend

The site‐average trend analysis approach informs us whether the overall riparian site (i.e., average of all the pixels within each riparian site) exhibits any trend in the NDVI data during the analysis period.

#### 3.1.2 Pixel‐based temporal trend

The pixel‐based analysis enabled us to assess whether there was any negative trend for each pixel (30 m × 30 m) in the riparian site. This analysis helped us to examine spatially explicit patterns of NDVI and the role of spatial proximity to croplands on vegetation health and diversity.

### 3.2 Cluster analysis

Cluster analysis examines the nature and strength of spatial relationships between adjacent features in space. This analysis was performed only for pixels that showed a negative NDVI trend. The results categorized pixels into 2 broad groups: random pixels and clustered pixels. Randomly distributed pixels lack a strong spatial correlation, which suggests a lack of common underlying factors responsible for the vegetation health decline. If there were a common underlying factor (e.g., exposure to herbicide drift) responsible for the observed decline, one would typically expect to find a cluster (or a plume) of negative pixels. Cluster analysis was performed using Spatial Autocorrelation (Morans I model) and Cluster and Outlier Analysis (Anselin Local Morans I model; Anselin 1995; Anselin et al. 2006) test available in ArcGIS10.3 (ESRI 2017). Clustered pixels were subjected to further analysis (proximity analysis, see *Statistical Analysis* section).

### 3.3 Proximity analysis

The distance of riparian zones to cropland is one of the key indicators of potential exposure from herbicide spray drift. Here, we looked at the distribution of clustered pixels relative to their distance from croplands.

#### 3.3.1 Downwind distance from croplands

Each riparian site was divided into 2 sections based on its location relative to the dominant wind direction and distance from croplands. The dominant wind direction in the April–May time period was chosen because herbicide applications in the Midwest and Great Plains are often made at or near the time of planting to prevent the spread and dominance of weeds before the crop has been established in the field (Abendroth et al. 2009). Distance of riparian sites to cropland plays an important role in potential impacts of herbicides on riparian zone vegetation, with herbicide drift deposition declining with increasing downwind distance (Gil et al. 2014; Kasiotis et al. 2014). If significant decline in riparian vegetation occurs mainly from spray drift, a spatial gradient within a riparian zone is expected to be evident in the NDVI data.

**Figure 2:**
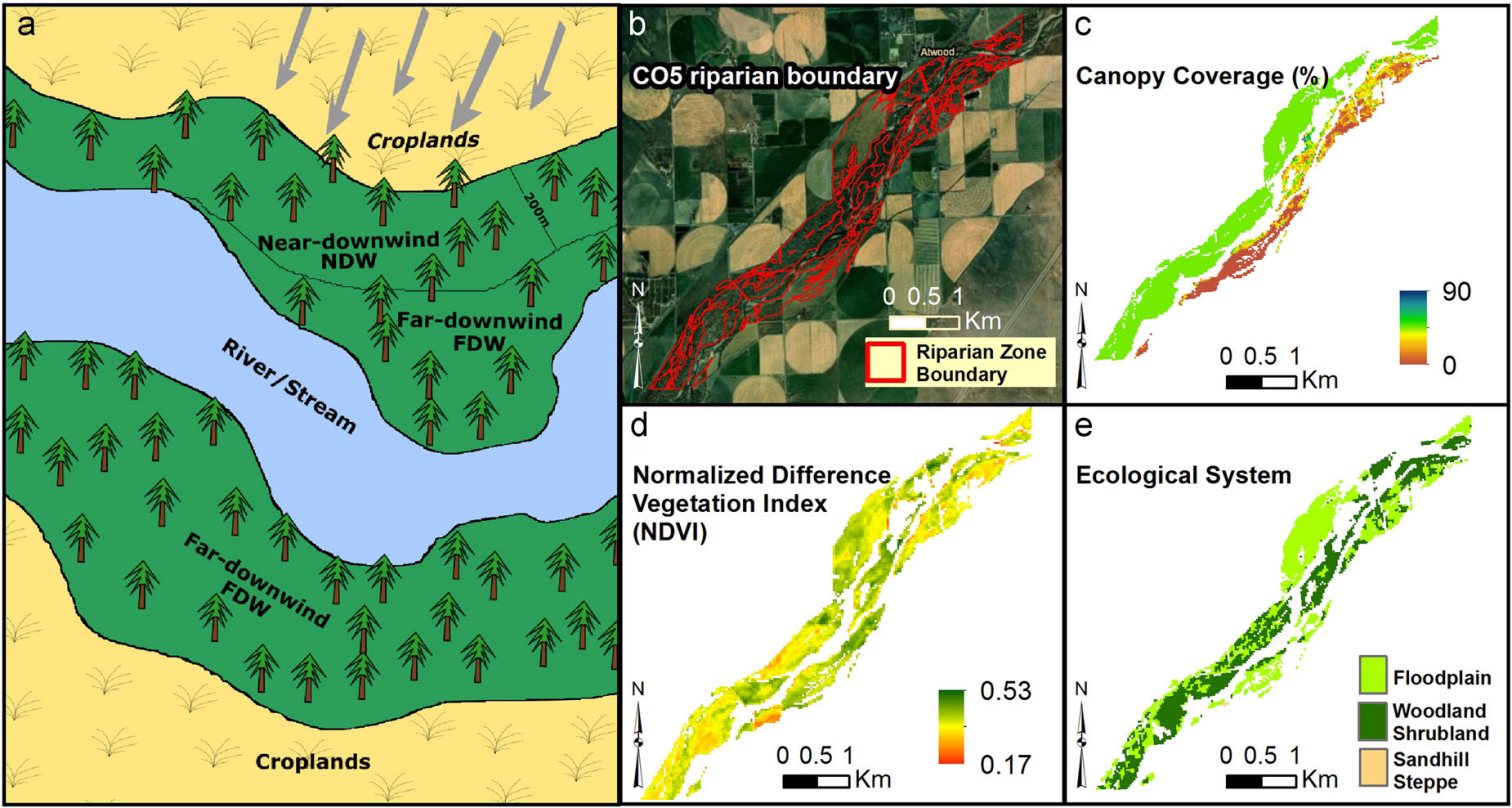
Schematics and actual data describing features of the riparian zone. Schematics of riparian zone where the dominant wind direction and distance from croplands defines the near‐downwind (NDW) versus far‐downwind (FDW) boundary (**a**); location of riparian zone and adjacent croplands at an example site (CO5) (**b**); canopy coverage (%) at each pixel at CO5 (**c**); average spring NDVI values at CO5 (**d**); ecological system present at each pixel at CO5 (**e**). CO5 = Colorado site 5).

We used 2 distance classes (Figure 2a): 1) the Near‐DownWind (NDW) class (0–200 m from the cropland), and 2) the Far‐DownWind (FDW) class (200m–400 m from the cropland). Riparian vegetation health and diversity were compared between these 2 distance classes. It is assumed that both NDW and FDW sections had similar biological (vegetation species, crop type, etc.) and climatic (wind speed, air temperature, precipitation, etc.) characteristics, such that the FDW riparian zone could effectively be used as a control. To investigate the sensitivity of our approach to the distance class definition, we performed the proximity analysis by varying the distance class definition where NDW is set to 0 to 50 m and FDW is set to 50 to 400 m.

#### 3.3.2 Near‐DownWind versus Far‐DownWind comparisons

We compared NDVI, CC, and VD between the NDW and FDW sections within each riparian site. We used the following statistics for comparison: the Welch 2‐sample *t*‐test (for CC) and the chi‐square distribution test (for VD).

## 4 RESULTS

### 4.1 Riparian site selection

#### 4.1.1 Site selection

A total of 17 riparian sites, which were considered potentially vulnerable to the effect of long‐term use of herbicides, were selected in the present study (see Figure 1). These sites comprise 5 sites in Colorado (CO1–CO5), 4 sites in Nebraska (NE1–NE4), 2 sites in Iowa (IA1–IA2), 2 sites in Missouri (MO1–MO2), and 4 sites in Kansas (KS1–KS4). These sites cover over a length of 680 km of riparian zone along major rivers, including the Platte River, Arkansas River, and Missouri River, and spread over an area of approximately 190 km^2^. All the selected sites were located inside the “high‐wind” regions, areas with high herbicide usage history, and were in the vicinity of croplands. Table 1 shows the location, length, and wind properties of these sites in greater detail. As shown in Table 1, the predominant wind direction was different across sites. Although some sites had a dominant northerly wind in April (NE1 and NE4), other sites had a dominant southerly pattern (KS2–KS4). For demonstration purposes, Figures 2b through 2e are presented to show the location of CO5 riparian site (as an example) in relation to croplands, its CC (%), long‐term average spring NDVI values, and ecological systems.

**Table 1:** Riparian site characteristics

Site name^a^	Site length (km)^b^	Area (km^2^)^b^	River or stream name	Avg wind direction^c^	Avg wind velocity (mph)^c^
CO1	40	7.2	South Platte River	N	11.1
CO2	40	5.6	South Platte River	N	11.1
CO3	33.6	11.8	South Platte River	NW	12.2
CO4	33.6	1.1	South Platte River	NW	12.2
CO5	33.6	4.1	South Platte River	N	13.5
IA1	25.6	12.3	Big Sioux River	N	11.8
IA2	46.4	14.3	Des Moines and East Des Moines rivers	NW	13.6
KS1	19.2	8	Arkansas River	N	12.2
KS2	27.2	5	Arkansas River	S	15
KS3	28.8	3	Arkansas River	S	15.3
KS4	83.2	4.1	Missouri River	SE	9.3
MO1	99.2	20.8	Missouri River	SE	9.9
MO2	80	3.4	Grand River	S	9.2
NE1	192	46.5	Platte River	N	13.5
NE2	192	10.5	Platte River	S	14.1
NE3	128	10.9	Platte River	S	14.1
NE4	64	8.7	Platte River	N	12.9

ASOS = Automated Surface Observing System; mph = miles per hour.

^a^ Site names are the combination of state abbreviation and site number (e.g., NE1 stands for Nebraska site 1).

^b^ Site length describes the length of the riparian zone along the known river or stream in kilometers (km), whereas area (km^2^) exhibits the total riparian zone area at each site.

^c^ Wind direction and wind velocities are dominant wind directions and average speed values for the month of April and May from the ASOS data set (1948–2011).

#### 4.1.2 General NDVI observations based on site‐average values

Both spatial and temporal variations were evident in NDVI values across the selected riparian sites (Table 2). The site‐average NDVI increased from south to north and from west to east during both spring and summer seasons (e.g., the average NDVI increased when moving from Colorado toward Nebraska and Iowa). The mean NDVI values increased at all sites as the season progressed toward summer with lowest values during April and highest values during July. Variation in the early spring values of NDVI were higher compared to summer months, with the greatest variation observed during May.

**Table 2:** General NDVI characteristics (mean and SD) at each riparian site for the duration of the study (1992–2011)^a^

Site^b^	Spring		Summer		April		May		June		July	
	Mean	SD	Mean	SD	Mean	SD	Mean	SD	Mean	SD	Mean	SD
CO1	0.40	0.06	0.53	0.09	0.33	0.05	0.47	0.07	0.53	0.09	0.54	0.09
CO2	0.38	0.05	0.55	0.08	0.29	0.04	0.44	0.06	0.54	0.07	0.56	0.08
CO3	0.37	0.05	0.52	0.07	0.29	0.04	0.44	0.06	0.51	0.07	0.53	0.07
CO4	0.37	0.05	0.54	0.07	0.28	0.04	0.45	0.06	0.53	0.07	0.55	0.07
CO5	0.37	0.04	0.54	0.07	0.29	0.04	0.45	0.06	0.53	0.07	0.55	0.07
IA1	0.48	0.08	0.79	0.07	0.29	0.04	0.62	0.11	0.73	0.10	0.81	0.06
IA2	0.43	0.05	0.79	0.07	0.26	0.04	0.64	0.09	0.77	0.08	0.81	0.05
KS1	0.34	0.04	0.56	0.07	0.23	0.02	0.44	0.06	0.52	0.07	0.58	0.07
KS2	0.35	0.05	0.44	0.08	0.27	0.04	0.44	0.08	0.43	0.07	0.46	0.09
KS3	0.35	0.05	0.46	0.07	0.29	0.04	0.40	0.06	0.46	0.08	0.46	0.07
KS4	0.57	0.10	0.77	0.11	0.44	0.08	0.71	0.13	0.77	0.11	0.78	0.11
MO1	0.51	0.10	0.69	0.10	0.34	0.07	0.62	0.13	0.68	0.11	0.71	0.10
MO2	0.52	0.12	0.70	0.15	0.39	0.08	0.65	0.16	0.69	0.16	0.70	0.15
NE1	0.47	0.06	0.67	0.08	0.30	0.05	0.53	0.07	0.67	0.08	0.68	0.08
NE2	0.48	0.07	0.71	0.07	0.34	0.07	0.60	0.08	0.68	0.08	0.74	0.07
NE3	0.50	0.05	0.76	0.07	0.32	0.05	0.66	0.07	0.74	0.07	0.77	0.06
NE4	0.54	0.04	0.78	0.05	0.34	0.03	0.69	0.06	0.77	0.06	0.79	0.05

NDVI = normalized difference vegetation index.

^a^ Results are presented at both seasonal and monthly levels.

^b^ Site names are the combination of state abbreviation and site number (e.g., NE1 stands for Nebraska site 1).

### 4.2 Trend analysis

#### 4.2.1 Site‐average temporal trend

**Figure 3:**
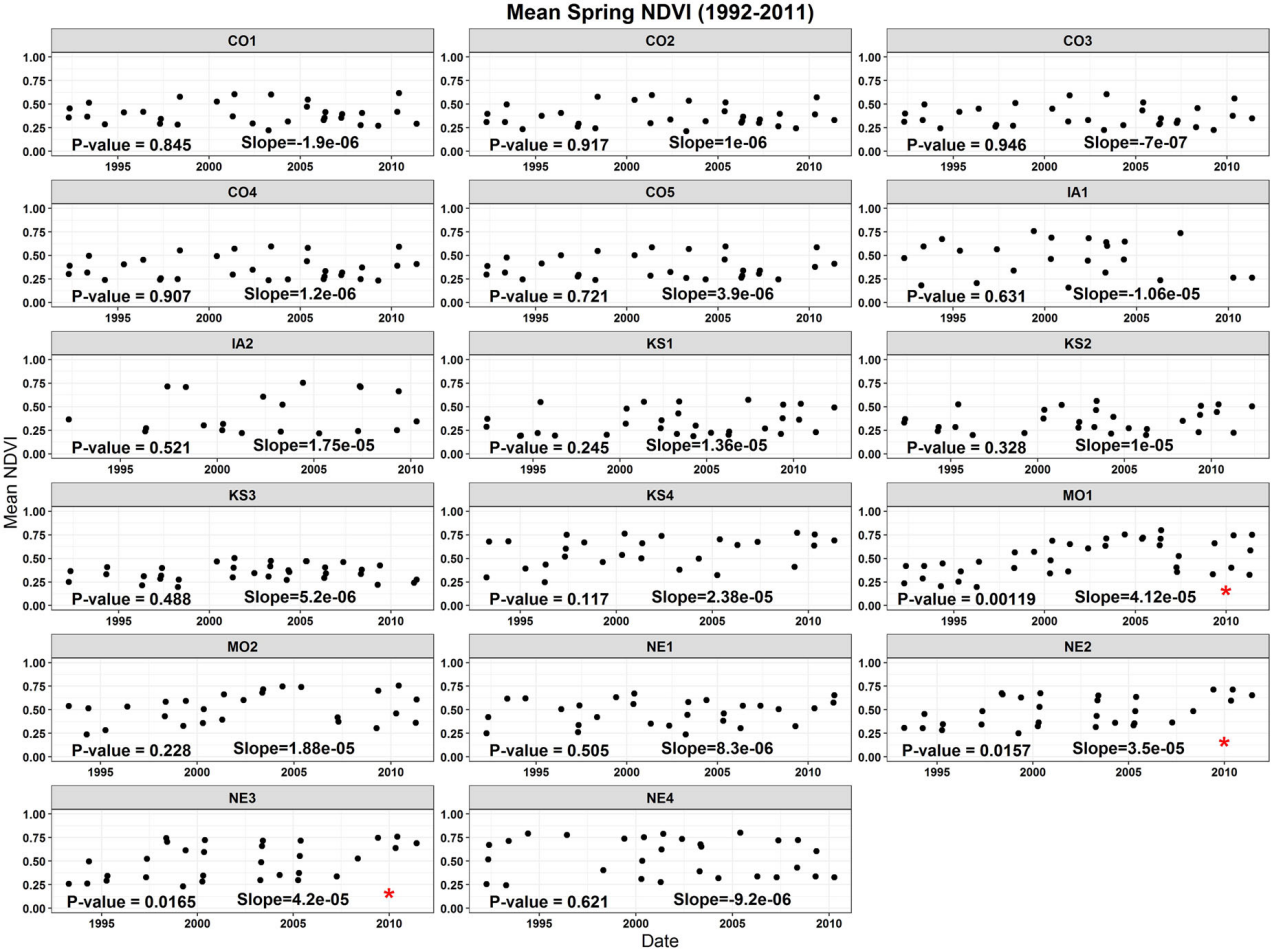
Site‐averaged NDVI time‐series for spring (April and May). Significance (*p*‐value) and the slope of the linear regression model are also presented for each site. Red asterisks represent sites that NDVI had a positive or negative trend. NDVI exhibited a positive trend at 3 sites (MO1, NE2, and NE3) between 1992 and 2011. Site names are abbreviated state names followed by a number (e.g., NE2 = Nebraska site 2, etc.). NDVI = normalized difference vegetation index.

**Figure 4:**
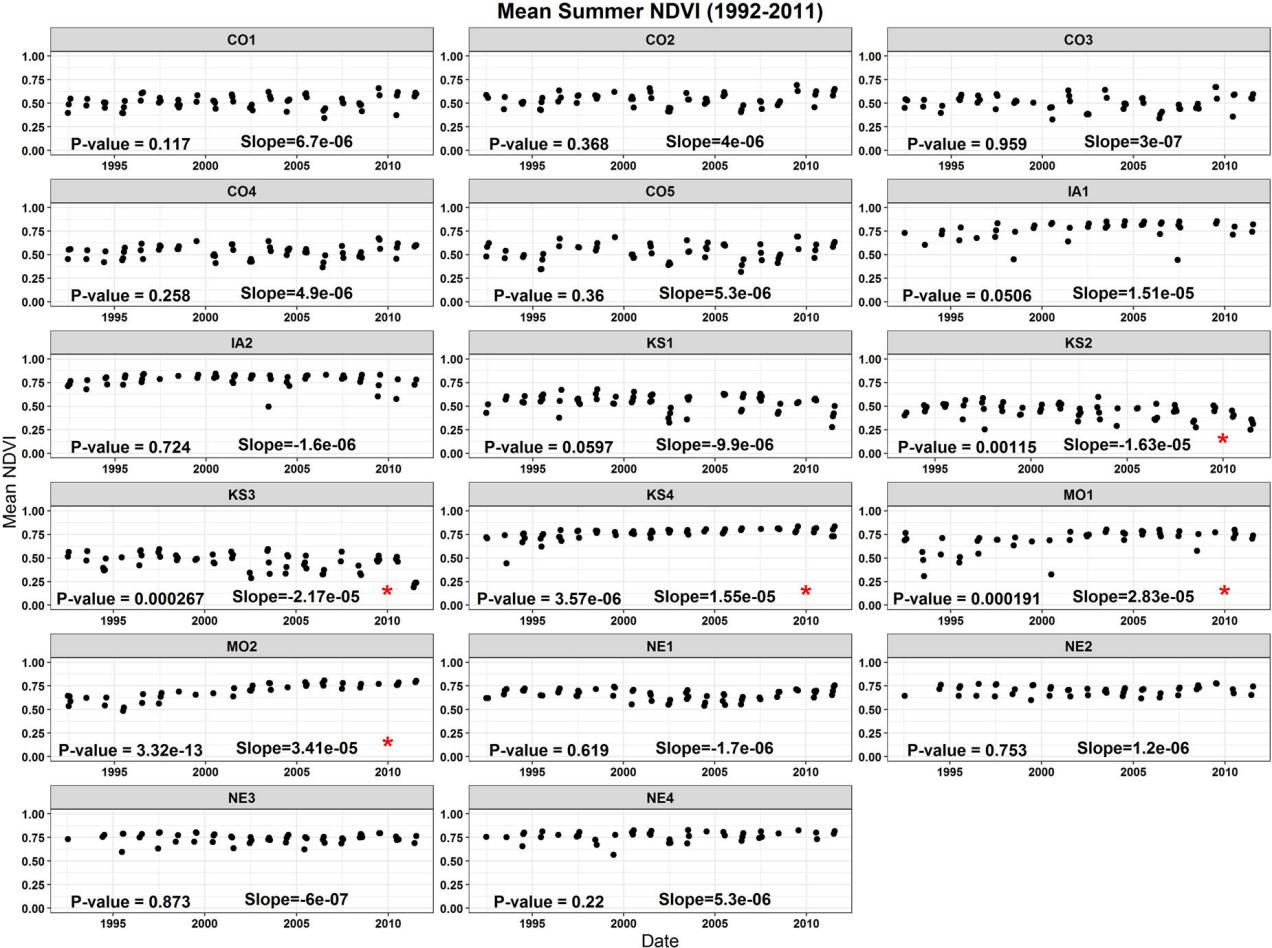
Site‐averaged NDVI time‐series for summer (June and July). Significance (*p*‐value) and the slope of the linear regression model is also presented for each site. Red asterisks represent sites that NDVI had a positive or negative trend. KS2 and KS3 both had negative NDVI trend, whereas KS4, MO1, and MO2 exhibited a positive trend. Site names are abbreviated state names followed by a number (e.g., KS2 = Kansas site 2, etc.). NDVI = normalized difference vegetation index.

The temporal trend (1992–2011) of the site‐averaged NDVI for all 17 sites was assessed at 2 levels (seasonal and monthly). Results are presented in Table 3 and in Figures 3 and 4. At the seasonal timescale, during the spring season (April and May), none of the 17 sites exhibited a negative trend for NDVI, whereas 3 sites (MO1, NE2, and NE3) showed a positive trend for NDVI (Figure 3). During the summer season (June and July), only 2 sites (KS2 and KS3) exhibited a negative trend for NDVI, whereas 3 sites exhibited a positive trend (KS4, MO1, and MO2; Table 3, Figure 4).

**Table 3:** Site‐averaged linear regression results for seasonal (left) and monthly (right) NDVI from 1992 to 2011

Site^a^	Spring		Summer		April		May		June		July	
	Slope	*p*‐value	Slope	*p*‐value	Slope	*p*‐value	Slope	*p*‐value	Slope	*p*‐value	Slope	*p*‐value
CO1	–1.88E–06	0.845	6.71E–06	0.117	1.89E–06	0.791	–3.08E–06	0.821	9.20E–06	0.143	3.40E–06	0.572
CO2	1.00E–06	0.917	3.97E–06	0.368	5.56E–06	0.358	–3.56E–07	0.977	2.79E–06	0.683	4.63E–06	0.411
CO3	–6.52E–07	0.946	2.54E–07	0.959	7.15E–07	0.898	1.91E–07	0.988	–1.14E–06	0.88	1.17E–06	0.857
CO4	1.22E–06	0.907	4.88E–06	0.258	2.38E–06	0.685	2.76E–06	0.844	4.87E–06	0.462	4.01E–06	0.470
CO5	3.89E–06	0.721	5.30E–06	0.36	5.07E–06	0.367	4.52E–06	0.752	3.09E–06	0.729	6.00E–06	0.406
IA1	–1.06E–05	0.631	1.51E–05	0.051	6.81E–06	0.706	2.16E–05	0.181	8.42E–06	0.58	1.82E–05^b^	0.001^b^
IA2	1.75E–05	0.521	–1.55E–06	0.724	2.71E–06	0.734	3.27E–05	0.149	–5.13E–06	0.451	3.59E–06	0.297
KS1	1.36E–05	0.245	–9.88E–06	0.06	5.93E–06	0.356	1.50E–05	0.353	–1.29E–05	0.129	–7.09E–06	0.158
KS2	9.95E–06	0.328	–1.63E–05^b^	0.00^b^	1.14E–06	0.898	1.55E–05	0.222	–1.87E–05^b^	0.019^b^	–1.51E–05^b^	0.017^b^
KS3	5.23E–06	0.488	–2.17E–05^b^	0^b^	3.42E–06	0.748	–5.45E–07	0.945	–2.19E–05^b^	0.028^b^	–2.10E–05^b^	0.005^b^
KS4	2.38E–05	0.117	1.55E–05^b^	0^b^	3.05E–05	0.064	6.53E–06	0.314	1.25E–05^b^	0^b^	1.89E–05^b^	0.002^b^
MO1	4.12E–05^b^	0.001^b^	2.83E–05^b^	0^b^	2.62E–05^b^	0.01^b^	5.04E–05^b^	0^b^	3.29E–05^b^	0.001^b^	2.57E–05^b^	0.024^b^
MO2	1.88E–05	0.228	3.41E–05^b^	0^b^	2.17E–05^b^	0.087^b^	4.11E–05^b^	0^b^	3.61E–05^b^	0^b^	3.22E–05^b^	0^b^
NE1	8.32E–06	0.505	–1.71E–06	0.619	1.41E–05	0.212	2.28E–06	0.837	–4.72E–06	0.303	1.06E–06	0.826
NE2	3.50E–05^b^	0.016^b^	1.17E–06	0.753	1.27E–05	0.077	1.26E–05	0.396	1.79E–06	0.663	3.41E–06	0.454
NE3	4.20E–05^b^	0.016^b^	–5.77E–07	0.873	1.72E–05	0.1	1.40E–05	0.356	3.59E–06	0.456	–3.52E–06	0.392
NE4	–9.15E–06	0.621	5.27E–06	0.22	8.00E–06	0.454	–5.50E–06	0.671	5.78E–06	0.45	2.02E–06	0.647

NDVI = normalized difference vegetation index.

^a^ Site names are the combination of state abbreviation and site number (e.g., NE1 stands for Nebraska site 1).

^b^ Values exhibit significant slope (*p*‐value ≤ 0.05); notice the slope sign for positive or negative trends.

At the monthly timescale, the results are overall comparable with those at the seasonal timescale. The monthly results reveal the following: During April and May, none of the 17 sites showed a negative trend, whereas 1 site in April (MO1) and 2 sites in May (MO1 and MO2) showed a positive trend; during June and July, only 2 sites showed a negative trend (KS2 and KS3).

#### 4.2.2 Pixel‐based temporal trend

Results of pixel‐based trend analysis of NDVI for spring and summer seasons are presented in Supplemental Data Figures S2 and S3, respectively. During the spring season, the NDVI did not change for the vast majority of sites, except at 4 sites (CO1, CO3, CO5, KS3), which had only 1% to 2% of their pixels showing a negative trend. In contrast, many sites had a considerable number of pixels with a positive trend (e.g., NE3 had greater than 93%, Supplemental Data Figure S2) during the spring season.

During summer, however, the percentage of pixels showing both negative and positive trends increased. Twelve sites (out of 17 total) had significant number of pixels (i.e., >1% of total number of pixels) showing negative‐trending NDVI (see Supplemental Data Figure S3). For these 12 sites, the percent of number of pixels with negative‐trending NDVI (out of the total number of pixels) is as follows (in decreasing order): KS1 = 28.7%, KS3 = 23.8%, KS2 = 18.9%, NE3 = 6.8%, NE1 = 5.3%, CO1 = 4.7%, CO3 = 4.0%, NE2 = 3.8%, IA2 = 3.2%, CO4 = 3.0%, IA1 = 1.1%, and NE4 = 1.0%. These sites underwent further analysis to detect whether those pixels were randomly distributed or clustered in space.

### 4.3 Cluster analysis

**Figure 5:**
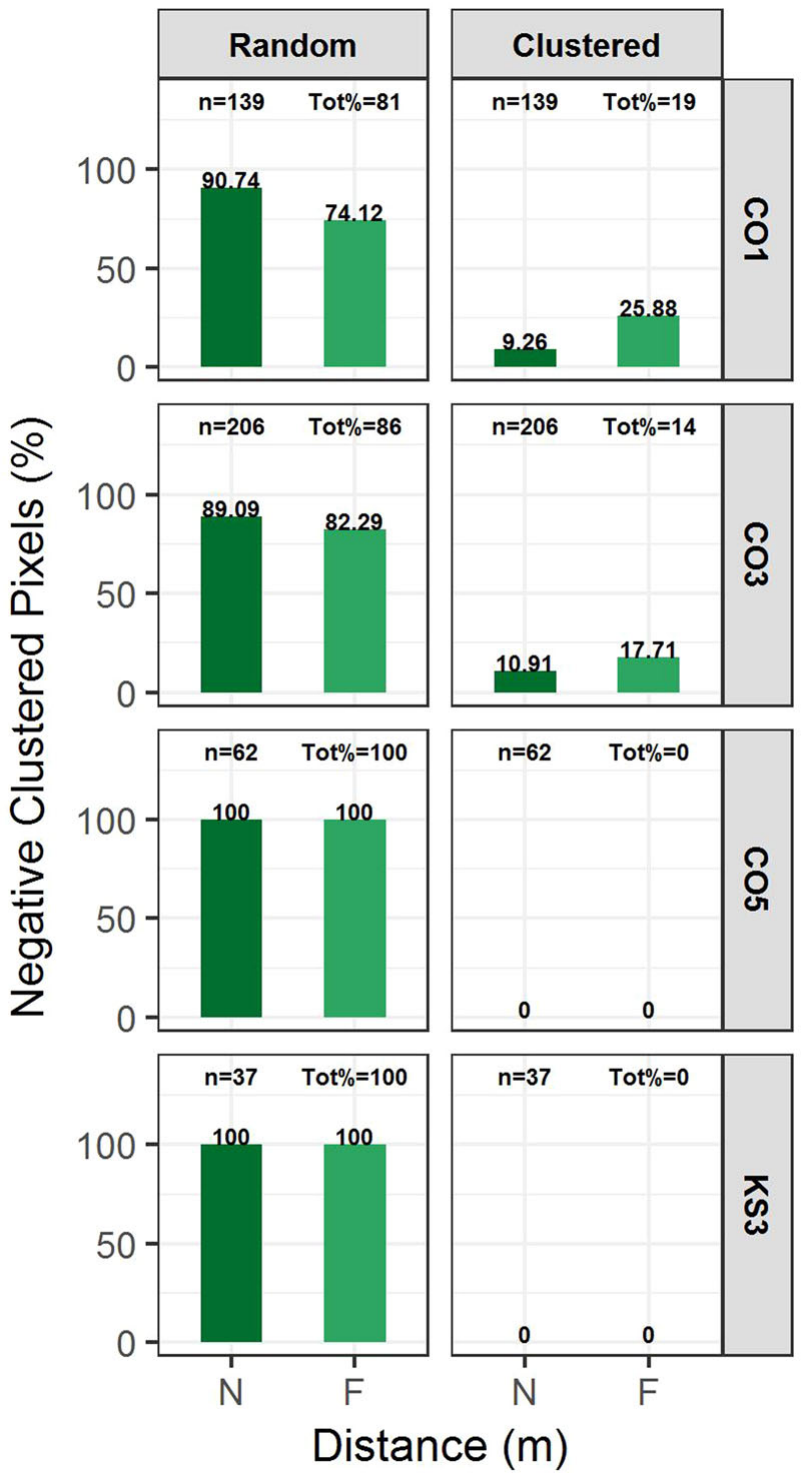
Cluster‐analysis results for the negative trending NDVI pixels during spring season. For each panel, the top titles exhibit the type of negative cluster whereas the vertical headings (right side) display the site name. Bars show the distribution (%) of clusters in various distance (*N* = 0–200 m, *F* = 200–400 m) from the croplands; *n* represents the total number of negative pixels carried over from pixel‐based trend analysis; Tot% presents the proportion of pixels in each category relative to *n*. This analysis was performed for 4 sites with greater than 1% negative pixels (CO1, CO3, CO5, and KS3). Site names are abbreviated state names followed by a number (e.g., CO1 = Colorado site 1, etc.). NDVI = normalized difference vegetation index.

**Figure 6:**
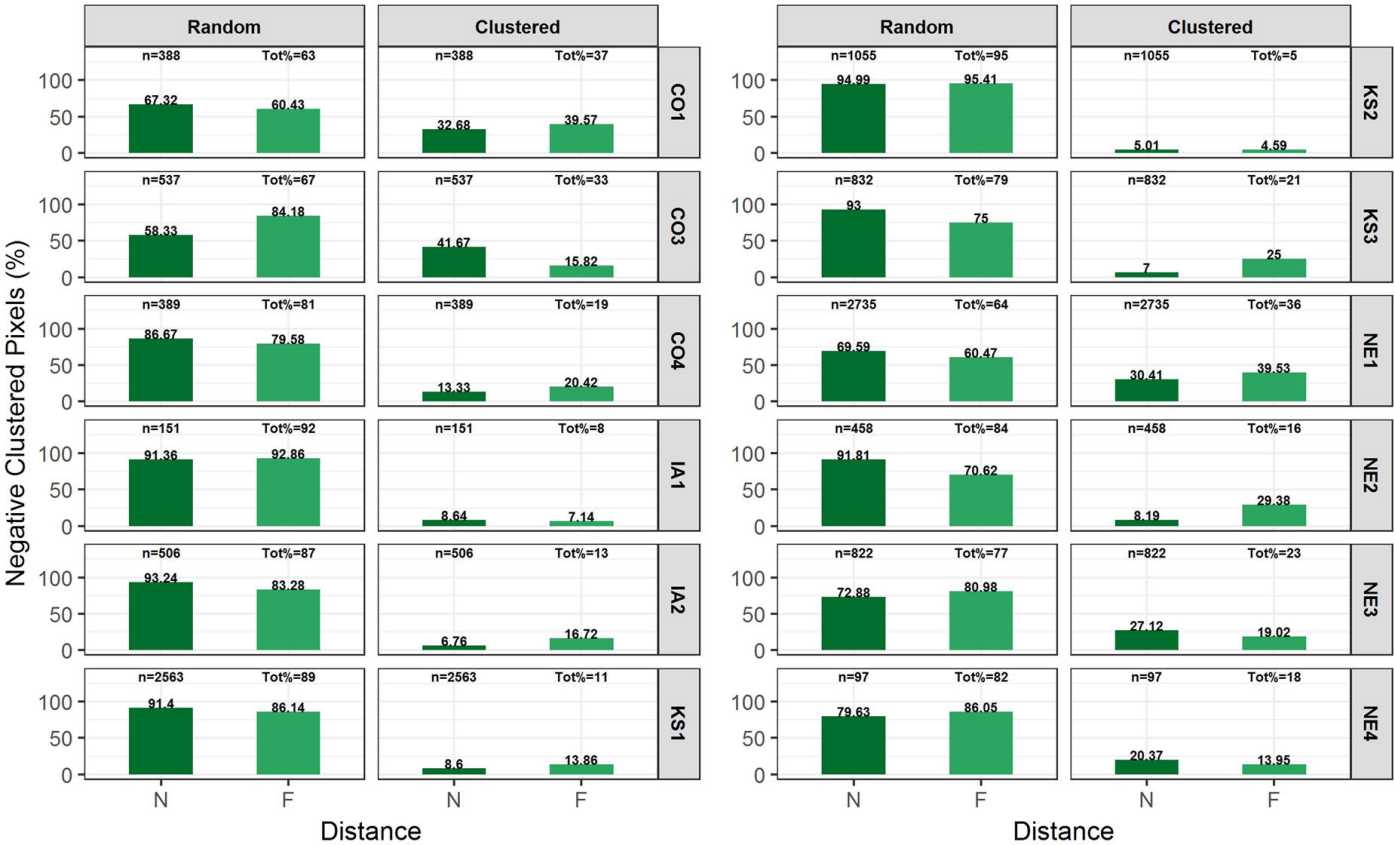
Cluster‐analysis results for the negative trending NDVI pixels during summer season. For each panel, the top titles exhibit the type of negative cluster whereas the vertical headings (right side) display the site name. Bars show the distribution (%) of clusters in various distance (*N* = 0–200 m, *F* = 200–400 m) from the croplands; *n* represents the total number of negative pixels carried over from pixel‐based trend analysis; Tot% presents the proportion of pixels in each category relative to *n*. This analysis was performed for 12 sites that had greater than 1% negative pixels (CO1, CO3, CO4, KS1–KS3, IA1–IA2, and NE1–NE4). Site names are abbreviated state names followed by a number (e.g., CO1 = Colorado site 1, etc.). NDVI = normalized difference vegetation index.

Sites with a significant number of negative‐trending pixels (i.e., >1% of total number of pixels) underwent rigorous cluster (or spatial autocorrelation) analysis (see Figures 5 and 6). These sites are 4 sites during spring (CO1, CO3, CO5, and KS3; Figure 5) and 12 sites during summer season (CO1, CO3, CO4, IA1, IA2, KS1, KS2, KS3, NE1, NE2, NE3, and NE4; Figure 6).

During both spring and summer seasons, the vast majority of the pixels showing negative trends in NDVI were randomly distributed in space. The proportion of analyzed negative‐trending pixels that were found to be clustered in spring season were 19% and 14% (CO1, CO3) and 0% (CO5 and KS3), accounting for less than 0.3% of the total pixels in each riparian site. During summer, the percent of negative‐trending NDVI pixels that were found clustered was 37% (CO1), 36% (NE1), 33% (CO3), 23% (NE3), 21% (KS3), 19% (CO4), 18% (NE4), 16% (NE2), 13% (IA2), 11% (KS1), 8% (IA1), and 5% (KS2). During summer, the clustered negative‐trending NDVI pixels accounted for only less than 5% of the total pixels in each riparian site: 5% for KS3, 3.2% for KS1, 1.9% for NE1, 1.8% for CO1, 1.6% for NE3, 1.3% for CO3, and less than 1% for the remaining sites analyzed (CO4, IA1, IA2, KS2, NE2, and NE4).

### 4.4 Proximity analysis

#### 4.4.1 Normalized difference vegetation index

The clustered negative‐trending NDVI pixels in both spring and summer seasons were distributed about equally in both NDW and FDW sections of the riparian site (Figures 5 and 6). Regardless of selected distance for NDW (0–50 m or 0–200 m), there was no indication that the NDW riparian pixels had higher percentages of clustered negative pixels than the FDW pixels (Supplemental Data Figure S4).

#### 4.4.2 Canopy coverage

Changes in CC were assessed by computing the difference map between the 2001 and the 2011 CC maps. In general, the average CC decreased in 2011, for both NDW and FDW sites. The average CC for NDW decreased from 37.1% in 2001 to 32.0% in 2011 (–5.2%). Similarly, the average CC for FDW decreased from 36.1% in 2001 to 31.7% in 2011 (–4.5%). Using the Welch 2‐sample *t*‐test, we compared the median CC change between NDW and FDW sites, and found out that the CC change between 2001 and 2011 was not significantly different (*t* = 1.77, *df* = 1581, *p* = 0.08) for both NDW and FDW sites (Table 4).

**Table 4:** Mean percent canopy coverage (%) change from 2001 to 2011 in the FDW and NDW sections at each riparian site

Site^a^	FDW^bc^ ^e^	NDW^bd^ ^e^	*p*–value
CO1	–1.49 (±11.4)	–3.3 (±11.2)	0
CO2	–2.56 (±10.4)	–0.82 (±10)	0.240
CO3	–3.28 (±10.7)	–3.92 (±10.4)	0.005
CO4	–0.04 (±8.3)	–3.59 (±11)	0.009
CO5	–1.33 (±9.3)	–2.44 (±10.7)	0.001
IA1	4.91 (±10.4)	6.59 (±10)	0.386
IA2	–6.74 (±15.4)	–8.76 (±14.8)	0.019
KS1	–5.75 (±20)	–8.7 (±17.5)	0
KS2	5.22 (±9.9)	4.23 (±9.4)	0.008
KS3	7.07 (±9.2)	5.52 (±7.1)	0
KS4	–5.39 (±19.5)	–2.9 (±16.6)	0.097
MO1	–4.41 (±18.6)	–5.44 (±20.4)	0.007
MO2	–6.79 (±20.9)	–3.43 (±21.1)	0.197
NE1	–6.8 (±13.2)	–9.04 (±15.1)	0
NE2	–17.15 (±12.6)	–10.81 (±14.8)	0.568
NE3	–15.05 (±14.6)	–18.87 (±13.9)	0.021
NE4	–16.39 (±15.9)	–22 (±17.8)	0

^a^ Site names are the combination of state abbreviation and site number (e.g., NE1 stands for Nebraska site 1).

^b^ Standard deviations are reported in parenthesis.

^c^ FDW represents the Far‐DownWind section (200–400 m from the cropland).

^d^ NDW represents Near‐DownWind section (0–200 m from the cropland) taking dominant wind direction as a reference.

^e^ Negative percent canopy coverage change values represent decreased canopy coverage between 2001 and 2011, whereas positive values exhibit increase in canopy coverage for the same period.

#### 4.4.3 Vegetation diversity proximity analysis

Vegetation diversity represents the number of existing vegetation groups at each riparian site. Vegetation diversity was compared across NDW and FDW sections. The chi‐square test result suggests that VD is not significantly different (*p* = 0.22) between the NDW and FDW sections. The dominant vegetation forms at both NDW and FDW sections were from the Southeastern Great Plains Floodplain Forest ecological group, followed by Western Great Plains Riparian Woodland and Shrubland (Table 5).

**Table 5:** Ecological groups present at each riparian region

Site^a^	Dominant ecosystem type^b^	Diversity^c^	Site	Dominant ecosystem type^b^	Diversity^c^
CO1 F	WGPRWS	5	KS2 N	WGPFS	8
CO1 N	WGPRWS	4	KS3 F	WGPFS	7
CO2 F	WGPF	5	KS3 N	WGPSP	7
CO2 N	WGPF	4	KS4 F	SGPFF	7
CO3 F	WGPRWS	6	KS4 N	SGPFF	8
CO3 N	WGPRWS	6	MO1 F	SGPFF	8
CO4 F	WGPF	7	MO1 N	SGPFF	9
CO4 N	WGPRWS	6	MO2 F	SGPFF	7
CO5 F	WGPRWS	3	MO2 N	SGPFF	6
CO5 N	WGPF	3	NE1 F	WGPFS	7
IA1 F	NCIMBF	11	NE1 N	WGPFS	8
IA1 N	SGPFF	11	NE2 F	WGPFS	8
IA2 F	SGPFF	10	NE2 N	WGPFS	7
IA2 N	SGPFF	6	NE3 F	SGPFF	7
KS1 F	WGPFS	8	NE3 N	SGPFF	7
KS1 N	WGPFS	9	NE4 F	SGPFF	3
KS2 F	WGPFS	8	NE4 N	SGPFF	4

F = Far; N = Near; NCIMBF = North‐Central Interior Maple‐Basswood Forest; SGPFF = Southeastern Great Plains Floodplain Forest; WGPF = Western Great Plains Floodplain; WGPFS = Western Great Plains Floodplains Systems; WGPRWS = Western Great Plains Riparian Woodland and Shrubland; WGPSP = Western Great Plains Shortgrass Prairie.

^a^ Site names are the combination of state abbreviation and site number (e.g., CO1 stands for Colorado site 1).

^b^ Dominant ecosystem type represents the most abundant ecological group at each site.

^c^ Diversity represents the number of ecological groups present at each riparian site.

## 5 DISCUSSION

Riparian zones host a variety of plant and animal species. Although many of these species are temporary residents of these habitats (e.g., insects, birds), others are year‐round residents (e.g., plant species, beavers). These ecosystems may also host some of the endangered plant species (Nilsson and Berggren 2000). Riparian sites also act as important biofilters in both stabilizing the river bank against erosion and reducing the inflow of nutrients into river ecosystems (Poff et al. 2011). Although a few federal and state provisions are in place to protect these ecosystems from destruction, many factors (urban development, mining, agriculture, etc.) could adversely impact these systems. Among these factors, little is known about the impacts on vegetation health and diversity of riparian ecosystems resulting from repeated exposures to off‐target agricultural herbicide drift.

Chemical herbicides are applied regularly for controlling the population of harmful weeds in croplands. Riparian zones in close proximity to agricultural fields are potentially vulnerable to drift exposures. In the present study, we evaluated the vegetation health and diversity of riparian sites near croplands with heavy herbicide usage history during the time period of 1992 to 2011. The riparian sites selected in the present study were located in areas of consistent wind speeds of more than 9 mph during the months of April and May, when herbicide applications are expected to be most intense (Table 1).

We relied on satellite remote sensing data collected during a 20‐y period (1992–2011) for the present study. Application of remote sensing data to assess vegetation health and diversity is common (Henry et al. 2004; Kogan et al. 2004; Dicke et al. 2012). We employed NDVI as the measure of vegetation health in riparian ecosystems. This simple index quantifies the vegetation greenness, which is often indicative of vegetation health. The NDVI values range between –1 and +1, and it is widely accepted that values between 0 and 0.02 primarily represent bare soil, whereas values greater than 0.2 are indicative of higher vegetation density. The NDVI values between 0.5 and 0.7 depict dense photosynthetically active vegetation (Holben 1986).

Judging from the average NDVI values, vegetation density was moderate (NDVI = 0.4–0.5) to high (NDVI = 0.6–0.8) at the 17 selected riparian sites. The average NDVI values increased from spring to summer at all of these sites, as expected. This is attributed to availability of favorable growth conditions (increases in ambient temperature, solar radiation, nutrient availability, etc.) and vegetation phenology. Sites at higher latitudes (IA1 and IA2 in Iowa) showed higher greenness values than did the southern sites (e.g., KS1) during both seasons. The dominant vegetation type varies between sites from hardwood woodland and shrubland species to shortgrass prairie species. Hardwood species tend to have a longer growing season than shortgrass prairie species. Therefore, some of the variability of NDVI values among sites could be attributed to differences in vegetation type and local climate. Nonetheless, long‐term trend in vegetation health at each site could potentially point at the role of external stressors in these systems.

### 5.1 Vegetation health

#### 5.1.1 Normalized difference vegetation index trend during the spring season

Over the study period, riparian vegetation health in the spring season remained unchanged in the majority of our study sites (14 sites out of 17), with 3 sites showing a positive trend in NDVI. The spring season is generally when most agricultural activities are resumed. This is also the season for heavy application of herbicides because many farmers prefer to control and enable their crops to outcompete weeds and unwanted shrubs early in the season (Abendroth et al. 2009). Although application dates vary between sites and years depending on local temperature and weather forecast, most herbicides are applied in the study region from mid and late April to early May (Abendroth et al. 2009). However, despite coinciding with the time frame in which potential off‐field herbicide drift is greatest, the overall spring trend of riparian vegetation health at these sites failed to show a statistically significant decline. Similar to patterns from the site‐average analysis, the majority of pixels at all sites had no remarkable trend during the spring season. The similarity between the 2 approaches (pixel‐level and site‐averaged analysis) confirms that the intrasite variability was not significant at these sites, and the site‐average–based analyses were able to capture the trends observed at smaller pixel‐by‐pixel scales.

#### 5.1.2 Normalized difference vegetation index trend during the summer season

In order to track any potential decline of riparian vegetation health due to delayed responses and/or late applications of herbicides, we also explored the trend of NDVI during the summer season. With the exception of 2 sites (KS2 and KS3), site‐averaged NDVI remained unchanged at the majority of our study sites (12 sites) and even increased at 3 sites (KS4, MO1, and MO2). The site‐averaged NDVI at KS2 decreased by 0.05 over the 20‐y period (was 0.4 in 1993 and 0.35 in 2011). A similar trend was observed at KS3 as well. We also carried out trend analysis at a pixel level (30 m × 30 m) in order to discern any spatially explicit patterns of long‐term trends. The pixel‐based analysis shows that 12 sites (out of 17) had significant number of pixels (>1% of the total number of pixels at each site) with negative‐trending NDVI. The large number of pixels with negative‐trending NDVI (>10% of total number of pixels) were observed for all the Kansas sites. The spatially explicit pixel‐by‐pixel analysis was found to be more robust than the site average for assessing spatial trends of riparian zone vegetation during the summer season.

#### 5.1.3 Spatial patterns of NDVI trends

In order to detect any localized impacts due to common underlying factors, such as drifted herbicides, we analyzed the spatial patterns of those pixels with negative‐trending NDVI. The spatial patterns examined are cluster analysis (whether the pixels were clustered around each other or randomly distributed in space) and proximity analysis (distance between the pixel and the cropland). The majority of these pixels were found to be randomly distributed in the space (i.e., unclustered) during both spring and summer seasons, indicating a low likelihood that the negative trends are in response to a common stressor. For example, for the 5 sites in Nebraska and Kansas (NE1, NE3, KS1, KS2, and KS3) that had 5% to 29% negative‐trending pixels during summer season, the negative‐trending pixels that were determined to be clustered ranged from about 0.1% (NE4) to 5% (at KS3) of the total number of pixels constituting riparian zone. For spring months, the proportion of negative‐trending pixels that were clustered (out of about 1% to 2% total negative‐trending pixels) also accounted for less than 0.3% of the total pixels.

At almost all sites, proportions of clustered negative pixels were similar between NDW (near) and FDW (far) sites (Figures 5 and 6). In other words, these clusters were “uniformly” distributed with respect to distance from croplands. It is assumed that both sections have similar biological (vegetation species, crop type, etc.) and climatic (wind speed, air temperature, precipitation, etc.) characteristics, such that the FDW riparian zone could effectively be used as a control. The available literature generally demonstrates a lack of statistically significant effects to in‐situ plant communities and individual plants at distances beyond the 6‐ to 10‐m range, even under worst‐case exposure paradigms (Marrs et al. 1989, 1991; Marrs and Frost 1997; Brain et al. 2017).

The negative‐trending NDVI pixels found in the present study for the 5 riparian sites in Nebraska and Kansas (NE1, NE3, KS1, KS2, and KS3) during summer season were intriguing; however, we could not find any significant association between the declining NDVI and distance to crop lands, suggesting that most of the negative trends were not likely caused by herbicide drifts. However, we must point out a caveat here regarding the limitation of our approach. The NDVI and remote sensing observations used in the present study did not allow us to exclusively isolate the impacts of herbicides from other potential factors that may affect the health of riparian vegetation. In addition, pixel‐based trend analysis of NDVI was limited to the available 30 m × 30 m scale, which may be insufficient to detect subpixel vegetation trends, structure, and diversity. Hence, additional work involving ecologically meaningful interpretation of negative trends and associated hydrological and management information is needed in order to gain a more complete picture of the conditions surrounding these ecosystems.

For instance, a regional shift in the hydrology of the Great Plains could offer some explanation for the observed negative trends. It is known that the riparian vegetation community composition will change in response to local and regional changes in hydrological conditions. River flow regimes and groundwater levels are among the 2 most important hydrological characteristics that are directly related to health and functioning of riparian ecosystems (Poff et al. 2011). For example, the multidecade‐long drought recorded from the early 1950s to the late 2000s and the consequent change in the river flow regime at the Arkansas River Basin (Eng and Brutsaert 1999; Putnam et al. 2008), where KS1, KS2, and KS3 sites are located, may be significant. In addition, the majority of sites in Nebraska (NE1, NE2, and NE3) and Kansas (KS1, KS2, and KS3) are located on the High Plains (HP) aquifer. A recent study has documented moderate to severe negative groundwater recharge (groundwater depletion) in central and southern parts of the HP aquifer, areas surrounding the Platte and Arkansas rivers where NE1, NE3, KS1, KS2, and KS3 sites are located (Scanlon et al. 2012). The lower groundwater recharge is directly translated to lower stream or river discharge (Scanlon et al. 2012), which could result in hydrologic stress on riparian vegetation in these regions (Lytle and Merritt 2004; Merritt et al. 2010).

### 5.2 Vegetation structure and diversity

Spatiotemporal analysis of CC revealed valuable information regarding the structure of vegetation at riparian sites between 2001 and 2011. On average, CC decreased in 2011 at almost all riparian sites. Based on the proximity test results, the average decrease in CC between NDW and FDW sections was not significantly different; that is, changes in percentage CC were equally distributed between both portions of each riparian site, and the patterns of CC loss appear to be independent of distance to upwind croplands.

Another striking result from the CC analysis is the positive increase of canopy in KS2 and KS3, in contrast to the decrease in general vegetation health during summer at these 2 sites. This divergence of results might arise from the fact that the NDVI does not distinguish between hardwood and shrubs. It is possible that the increase in CC is associated with an increase in the biomass of hardwood plants, whereas the NDVI is primarily influenced by the shrubs that dominate these 2 sites. Similar results have been reported in other studies (Serrat‐Capdevila et al. 2007; Seavy et al. 2009), in which slight climatic or hydrological variability reduced the population of shrubs in riparian ecosystems.

Looking at the composition of vegetation ecological groups at these riparian sites (GAP data set), the number of ecological groups was not significantly different between NDW and FDW sections. The dominant vegetation type, Southeastern Great Plains Floodplain Forest, was similar for both NDW and FDW sections of the sites. These riparian systems are floodplain forests that occupy relatively broad flats at low topographic locations, along large streams or rivers where alluvial deposition exists. Dominant communities in this system range from floodplain forests to wet meadows and gravel and sand flats. The second most dominant ecological group at these sites is the Western Great Plains Floodplain Systems. This group is found in the floodplains of medium to large rivers of the western Great Plains, where alluvial soils and periodic, intermediate flooding (every 5–25 y) is typical. The VD (GAP) data set was available only for the year 2001, so we could not examine the temporal variation of ecological groups within these sites. However, similarities in the type of dominant ecological group between the NDW and FDW sections of the riparian sites suggest relative stability in the vegetation community, with no remarkable difference in community structure of the riparian ecosystems with respect to their proximity to croplands.

## 6 SUMMARY AND CONCLUSIONS

We assessed the long‐term vegetation health and diversity of various riparian ecosystems in the vicinity of agricultural areas of the Midwest and Great Plains regions where there has been historically repeated and intensive use of herbicides. First, we identified the riparian sites that were potentially most vulnerable to drift of herbicides applied in agricultural areas. Our study sites (covering a total length of more than 680 km and an area of 190 km^2^) are comprised of 5 sites in Colorado, 4 sites in Nebraska, 2 sites in Iowa, 2 sites in Missouri, and 4 sites in Kansas. We used the NDVI, derived from Landsat satellite imageries, as indicator for vegetation health. Our methodology involved 1) trend analysis of NDVI using 20 y of monthly data; 2) spatial pattern (randomly distributed vs clustered) of NDVI trends; 3) variation of NDVI trend as a function of distance from cropland; 4) change in CC (derived from Landsat) between 2002 and 2011, and its dependence on distance from cropland; and 5) dependence of VD (derived from Landsat) on distance from cropland. We performed the analysis during both the spring season (when heavy application of herbicides takes places) and the summer season (to track delayed response or late application of herbicides). Our results can be summarized as follows:

1) During the spring season, the vegetation health status did not show any systematic change during the study period (1992–2011) at all the riparian sites.
2) During the summer season, the vegetation health did not show any systematic change during the study period for the vast majority of the riparian sites. However, a few exceptions were located within sites in Kansas and Nebraska.
3) For the few exceptional sites in Kansas and Nebraska, where NDVI shows a negative trend over time, we performed 2 additional analyses (cluster analysis and proximity analysis) to examine the nature of the factors and processes that may have caused the negative trend in NDVI and found the following:

- The vast majority of these NDVI pixels were randomly distributed through the riparian sites, suggesting the lack of localized underlying factor (such as drifted herbicide). The percentage of the pixels that were spatially clustered was small and ranged from 0.1% (a site in Nebraska) to 5% (a site in Kansas) of the total number of pixels constituting the riparian zones.
- The proximity analysis revealed that there was no association between the negative NDVI trend and distance of those pixels from croplands, suggesting that exposure to herbicide drift may not be a plausible factor because this would have shown higher impact on pixels closer to the cropland.
- The proximity analysis using additional data (CC and VD) also reported the lack of association between these vegetation characteristics and distance of pixels from cropland, again suggesting lack of evidence for the impact of herbicide exposure.

Based on our findings, we conclude the following:

1) The vast majority of the riparian ecosystem did not show any long‐term trend in their vegetation health, despite their potential exposure to historically repeated and intensive use of herbicides.
2) There are a few cases within the Kansas and Nebraska sites that showed a decline in vegetation health, particularly during the summer season; however, the spatial pattern of the trends is such that the exposure to herbicide use may not be a plausible cause.

Finally, we point out a caveat regarding the limitation of the present study. The NDVI index shows the combined impacts of all external stressors (e.g., herbicide, pests, water stress, erosion, deforestation) and does not isolate the impacts of herbicide use. Although we have looked at spatial pattern in an attempt to isolate the impacts of herbicide, further work is recommended to conduct field survey and isolate the impacts of herbicides at finer spatial resolution in order to corroborate the findings of the present study.

## Disclaimer

In accordance with *IEAM* policy and my ethical obligations, I would like to report that this research was funded by Syngenta Crop Protection. In preparation of this study, L Ghebremichael and J Perine from Syngenta helped with study design and reviewed the manuscript before submission to *IEAM*.

## Data Accessibility

We used available preprocessed data for this article. Surface wind speed data were collected from National Renewable Energy Laboratory (NREL, http://www.nrel.gov/), whereas monthly wind speed and direction was downloaded from Automated Surface Observing System (ASOS) stations (https://mesonet.agron.iastate.edu/). Landsat NDVI data were acquired from the EROS (Earth Resources Observation and Science) Science Processing Architecture (ESPA, https://espa.cr.usgs.gov/) repository. Tree canopy coverage data were downloaded from the National Land Cover Database (NLCD, http://www.mrlc.gov/index.php), and the Gap Analysis Program (GAP, http://gapanalysis.usgs.gov/) was used for detailed vegetation type distribution and change analysis.

## SUPPLEMENTAL DATA

**Figure S1.** Flowchart summarizing methods and procedures followed in this study.

**Figure S2.** Pixel‐based trend analysis of NDVI index for spring (April and May). NDVI = normalized difference vegetation index.

**Figure S3.** Pixel‐based trend analysis of NDVI index for summer (June and July). NDVI = normalized difference vegetation index.

**Figure S4.** Sensitivity analysis of cluster‐analysis for summer NDVI at different selected distances to croplands. NDVI = normalized difference vegetation index.

## Supporting information

This article includes online‐only Supplemental Data.

Supplemental Data.

## References

[ieam4144-bib-0001] Abendroth L , Elmore R , Hartzler R , McGrath C , Mueller D , Munkvold G , Pope R , Rice M , Robertson A , Sawyer J et al. 2009. Corn field guide. Ames (IA): Iowa State Univ Extension and Outreach Publications. 86 p.

[ieam4144-bib-0002] Andrews J , Fishburn E , Frazier B , Johnson R. 1985. North Fork John Day River habitat improvement: Annual report FY 1985. In: Natural Propagation and Habitat Enhancement, Volume I ‒ Oregon. Portland (OR): US Department of Energy, Bonneville Power Administration, Division of Fish and Wildlife. Project No. 84‐8. p 222‒261.

[ieam4144-bib-0003] Anselin L. 1995 Local indicators of spatial association‐LISA. Geogr Anal 27(2):93–115.

[ieam4144-bib-0004] Anselin L , Syabri I , Kho Y. 2006 GeoDa: An introduction to spatial data analysis. Geogr Anal 38(1):5–22.

[ieam4144-bib-0057] [ASOS] Automated Surface Observing System . 1998. ASOS User's Guide. Washington (DC): US Dept. of Commerce, National Oceanic and Atmospheric Administration; Federal Aviation Administration; US Navy; US Dept. of the Air Force.

[ieam4144-bib-0058] Baker NT , Stone WW . 2015 Estimated annual agricultural pesticide use for counties of the conterminous United States, 2008‒12. US Geological Survey Data Series 907. 9 p. 10.3133/ds907

[ieam4144-bib-0005] Bento VA , Gouveia CM , DaCamara CC , Trigo IF. 2018 A climatological assessment of drought impact on vegetation health index. Agric For Meteorol 259:286–295.

[ieam4144-bib-0006] Bowler DE , Mant R , Orr H , Hannah DM , Pullin AS. 2012 What are the effects of wooded riparian zones on stream temperature? Environ Evid 1(1):3.

[ieam4144-bib-0007] Brain RA , Perine J , Cooke C , Ellis CB , Harrington P , Lane A , O’Sullivan C , Ledson M. 2017 Evaluating the effects of herbicide drift on nontarget terrestrial plants: A case study with mesotrione. Environ Toxicol Chem 36(9):2465–2475. 28262983 10.1002/etc.3786

[ieam4144-bib-0008] Comer P , Faber‐Langendoen D , Evans R , Gawler S , Josse C , Kittel G , Menard S , Pyne M , Reid M , Schulz K et al. 2003. Ecological systems of the United States: A working classification of U.S. terrestrial systems. Arlington (VA): NatureServe. Technical report. 83 p.

[ieam4144-bib-0009] Coulston JW , Moisen GG , Wilson BT , Finco MV , Cohen WB , Brewer CK. 2012 Modeling percent tree canopy cover: A pilot study. Photogramm Eng Rem S 78(7):715–727.

[ieam4144-bib-0010] Dalton RL , Boutin C , Pick FR. 2015 Nutrients override atrazine effects on riparian and aquatic plant community structure in a North American agricultural catchment. Freshwater Biol 60:1292–1307.

[ieam4144-bib-0011] Dicke D , Jacobi J , Büchse A. 2012 Quantifying herbicide injuries in maize by use of remote sensing. *Julius‐Kuhn‐Archiv* 434:199‒205. doi: 10.5073/jka.2012.434.024

[ieam4144-bib-0059] Draxl C , Clifton A , Hodge BM , McCaa J . 2015 The Wind Integration National Dataset (WIND) toolkit. *Appl Energy* 151:355‒366.

[ieam4144-bib-0012] Eng K , Brutsaert W. 1999 Generality of drought flow characteristics within the Arkansas River Basin. J Geophys Res 104(1):435–441.

[ieam4144-bib-0013] [ESRI] Environmental Systems Research Institute . 2017 ArcGIS. https://www.esri.com/en-us/home

[ieam4144-bib-0014] Fensholt R , Sandholt I , Stisen S. 2006 Evaluating MODIS, MERIS, and VEGETATION vegetation indices using in situ measurements in a semiarid environment. IEEE Trans Geosci Remote Sens 44(7):1774–1786.

[ieam4144-bib-0015] Fierke MK , Kauffman JB. 2006 Invasive species influence riparian plant diversity along a successional gradient, Willamette River, Oregon. Nat Area J 26(4):376–382.

[ieam4144-bib-0016] Flores‐Cardenas F , Millan‐Aguilar O , Diaz‐Lara L , Rodriguez‐Arredondo L , Hurtado‐Oliva MA , Manzano‐Sarabia M. 2018 Trends in the normalized difference vegetation index for Magrove areas in Northwestern Mexico. J Coastal Res 34(4):877–882.

[ieam4144-bib-0017] Gil E , Balsari P , Gallart M , Llorens J , Marucco P , Andersen PG , Fàbregas X , Lop J. 2014 Determination of drift potential of different flat fan nozzles on a boom sprayer using a test bench. Crop Prot 56:58–68.

[ieam4144-bib-0018] Gregory SV , Swanson FJ , McKee WA , Cummins KW. 1991 An ecosystem perspective of riparian zones. BioScience 41(8):540–551.

[ieam4144-bib-0019] Grube A , Donaldson D , Kiely T , Wu L. 2011 Pesticides industry sales and usage: 2006 and 2007 market estimates. Washington (DC): US Environmental Protection Agency p 1–41.

[ieam4144-bib-0020] Hartley D , Kidd H. 1983 The agrochemicals handbook. Nottingham (UK): Royal Society of Chemistry. 400 p.

[ieam4144-bib-0021] Henry WB , Shaw DR , Reddy HR , Bruce LM , Tamhankar HD. 2004 Remote sensing to detect herbicide drift on crops. Weed Technol 18:358–368.

[ieam4144-bib-0022] Holben BN. 1986 Characteristics of maximum‐value composite images from temporal AVHRR data. Int J Remote Sens 7(11):1417–1434.

[ieam4144-bib-0023] Homer C , Dewit J , Fry J , Coan M , Hossai N , Larson C , Herold N , Mckerrow A , Vandriel JN , Wickham J. 2007 Completion of the 2001 National land cover database for the conterminous United States. Photogramm Eng Rem S 73:337–341.

[ieam4144-bib-0024] Howe WH , Knopf FL. 1991 On the imminent decline of Rio Grande cottonwoods in central New Mexico. Southwestern Nat 36(2):218–224.

[ieam4144-bib-0025] Inurreta‐Aguirre HD , Lauri PE , Dupraz C , Gosme M. 2018 Yield components and phenology of durum wheat in a Mediterranean alley‐cropping system. Agroforest Syst 92(4):961–974.

[ieam4144-bib-0026] Johnson MR , Zelt RB. 2005. Protocols for mapping and characterizing land use / land cover in riparian zones. Reston (VA): US Geological Survey. Open file report 2005‐1302. 22 p.

[ieam4144-bib-0027] Johnson RR , Carothers SW. 1982 Riparian habitats and recreation: Interrelationships and impacts in the Southwest and Rocky Mountain region. Eisen Consortium Bull 12(September):1–31.

[ieam4144-bib-0028] Kasiotis KM , Glass CR , Tsakirakis AN , Machera K. 2014 Spray drift reduction under Southern European conditions: A pilot study in the Ecopest Project in Greece. Sci Total Environ 479–480:132–137. 10.1016/j.scitotenv.2014.01.088 24561292

[ieam4144-bib-0029] Klink K. 1999 Climatological mean and interannual variance of United States surface wind speed, direction and velocity. Int J Climatol 19(5):471–488.

[ieam4144-bib-0030] Kogan F , Stark R , Gitelson A , Jargalsaikhan L , Dugrajav C , Tsooj S. 2004 Derivation of pasture biomass in Mongolia from AVHRR‐based vegetation health indices. Int J Remote Sens 25(14):2889–2896.

[ieam4144-bib-0031] Lewis DF , Jeffries MD , Gannon TW , Richardson RJ , Yelverton FH. 2014 Persistence and bioavailability of Aminocyclopyrachlor and Clopyralid in turfgrass clippings: Recycling clippings for additional weed control. Weed Sci 62(3):493–500.

[ieam4144-bib-0032] Lytle DA , Merritt DM. 2004 Hydrologic regimes and riparian forests: A structured population model for cottonwood. Ecology 85(9):2493–2503.

[ieam4144-bib-0033] Mariano DA , dos Santos CA. C , Wardlow BD , Anderson MC , Schiltmeyer AV , Tadesse T , Svoboda MD. 2018 Use of remote sensing indicators to assess effects of drought and human‐induced land degradation on ecosystem health in Northeastern Brazil. Remote Sens Environ 213:129–143.

[ieam4144-bib-0035] Marrs R , Frost A , Plant R. 1991 Effect of mecoprop drift on some plant species of conservation interest when grown in standardized mixtures in microcosms. Environ Pollut 73(1):25–42. 15092089 10.1016/0269-7491(91)90094-d

[ieam4144-bib-0034] Marrs RH , Frost AJ. 1997 A microcosm approach to the detection of the effects of herbicide spray drift in plant communities. J Environ Manage 50(4):369–388.

[ieam4144-bib-0036] Marrs RH , Williams CT , Frost AJ , Plant RA. 1989 Assessment of the effects of herbicide spray drift on a range of plant species of conservation interest. Environ Pollut 59(1):71–86. 15092416 10.1016/0269-7491(89)90022-5

[ieam4144-bib-0037] Martin DE , Latheef MA. 2017 Remote sensing evaluation of two‐spotted spider mite damage on greenhouse cotton. J Visual Exp 122:e54314. 10.3791/54314 PMC5565137 28518067

[ieam4144-bib-0038] Martin TL , Kaushik NK , Trevors JT , Whiteley HR. 1999 Review: Denitrification in temperate climate riparian zones. Water, Air, Soil Pollut 111:171–186.

[ieam4144-bib-0041] Mashaba Z , Chirima G , Botai J , Combrinck L , Munghemezulu C. 2016 Evaluating spectral indices for winter wheat health status monitoring in Bloemfontein using Lsat 8 data. S Afr J Geomatics 5(2):227–243.

[ieam4144-bib-0039] Mazzarino M , Finn JT. 2016 An NDVI analysis of vegetation trends in an Andean watershed. Wetlands Ecol Manage 24(6):623–640.

[ieam4144-bib-0040] Merritt DM , Scott ML , Leroy Poff N , Auble GT , Lytle DA. 2010 Theory, methods and tools for determining environmental flows for riparian vegetation: Riparian vegetation‐flow response guilds. Freshwater Biol 55(1):206–225.

[ieam4144-bib-0043] NatureServe . 2017 NatureServe Explorer: An online encyclopedia of life. Version 7.1. Arlington (VA). http://explorer.natureserve.org

[ieam4144-bib-0044] Nilsson C , Berggren K. 2000 Alterations of riparian ecosystems caused by river regulation. BioScience 50(9):783.

[ieam4144-bib-0045] Niman RJ , Decamps H , Pollock M. 1993 The role of riparian corridors in maintaining regional biodiversity. Ecol Appl 3(2):209–212. 27759328 10.2307/1941822

[ieam4144-bib-0046] Obedzinski R , Shaw C , Neary D. 2001 Declining woody vegetation in riparian ecosystems of the western United States. West J Appl For 16:169–181.

[ieam4144-bib-0047] Poff B , Koestner KA , Neary DG , Henderson V. 2011 Threats to riparian ecosystems in Western North America: An analysis of existing literature. J Am Water Resour As 47(6):1241–1254.

[ieam4144-bib-0048] Prakash NR , Singh RK , Chauhan SK , Sharma MK , Bharadwaj C , Hegde VS , Jain PK , Gaur PM , Tripathi S. 2017 Tolerance to post‐emergence herbicide imazethapyr in chickpea. Indian J Genet Pl Br 77(3):400–407.

[ieam4144-bib-0049] Putnam JE , Perry CA , Wolock DM. 2008. Hydrologic droughts in Kansas ‐ Are they becoming worse? Rolla (MO): Rolla Publ Service Ctr. USGS Fact Sheet 2008‐3034. 6 p.

[ieam4144-bib-0050] Scanlon BR , Faunt CC , Longuevergne L , Reedy RC , Alley WM , McGuire VL , McMahon PB. 2012 Groundwater depletion and sustainability of irrigation in the US High Plains and Central Valley. Proc Natl Acad Sci USA 109(24):9320–9325. 22645352 10.1073/pnas.1200311109 PMC3386121

[ieam4144-bib-0051] Seavy N. E , Gardali T , Golet GH , Griggs FT , Howell CA , Kelsey R , Small SL , Viers JH , Weigand JF. 2009 Why climate change makes riparian restoration more important than ever: Recommendations for practice and research. Ecol Restor 27(3):330–338.

[ieam4144-bib-0052] Serrat‐Capdevila A , Valdés JB , Pérez JG , Baird K , Mata LJ , Maddock T. 2007 Modeling climate change impacts ‐ and uncertainty ‐ on the hydrology of a riparian system: The San Pedro Basin (Arizona/Sonora). J Hydrol 347(1‐2):48–66.

[ieam4144-bib-0053] Spera DA , Richards TR. 1979. Modified power law equations for vertical wind profiles. Cleveland (OH): National Aeronautics and Space Administration, Lewis Research Center. p 1–12.

[ieam4144-bib-0054] Thelen KD , Kravchenko AN , Lee CD. 2004 Use of optical remote sensing for detecting herbicide injury in soybean. Weed Technol 18:292–297.

[ieam4144-bib-0055] Thelin GP , Stone WW. 2013. Estimation of annual agricultural pesticide use for counties of the conterminous United States, 1992‐2009. Reston (VA): USGS. Technical report. 54 p.

[ieam4144-bib-0042] US Fish and Wildlife Service . 2017 National Wetlands Inventory website. Washington (DC): US Department of the Interior, Fish and Wildlife Service. http://www.fws.gov/wetlands/

[ieam4144-bib-0056] Van Leeuwen WJD , Hartfield K , Miranda M , Meza FJ. 2013 Trends and ENSO/AAO driven variability in NDVI derived productivity and phenology alongside the Andes Mountains. Remote Sens 5(3):1177–1203.

