# Supplementary material for: Remote‐sensing Based Assessment of Long‐term Riparian Vegetation Health in Proximity to Agricultural Lands with Herbicide Use History
Source: Integr Environ Assess Manag. 2019 Jun 22;15(4):528–43. doi: 10.1002/ieam.4144 (PMC6852167; doi:10.1002/ieam.4144)
Supplement: Supplementary file 1 — Supplemental Data. [file IEAM-15-528-s001.docx]

**SUPPLEMENTARY MATERIAL**

**Title of article:**

Remote-sensing Based Assessment of Long-term Riparian Vegetation Health in Proximity to Agricultural Lands with Herbicide Use History

**Authors:**

Foad Yousef^1^, Mekonnen Gebremichael^1^, Lula Ghebremichael^2^ and Jeffrey Perine^2^

**Affiliations:**

^1^Department of Civil and Environmental Engineering, University of California Los Angeles, Los Angeles, CA, 90095

^2^Syngenta Crop Protection LLC., Greensboro, NC, 27409


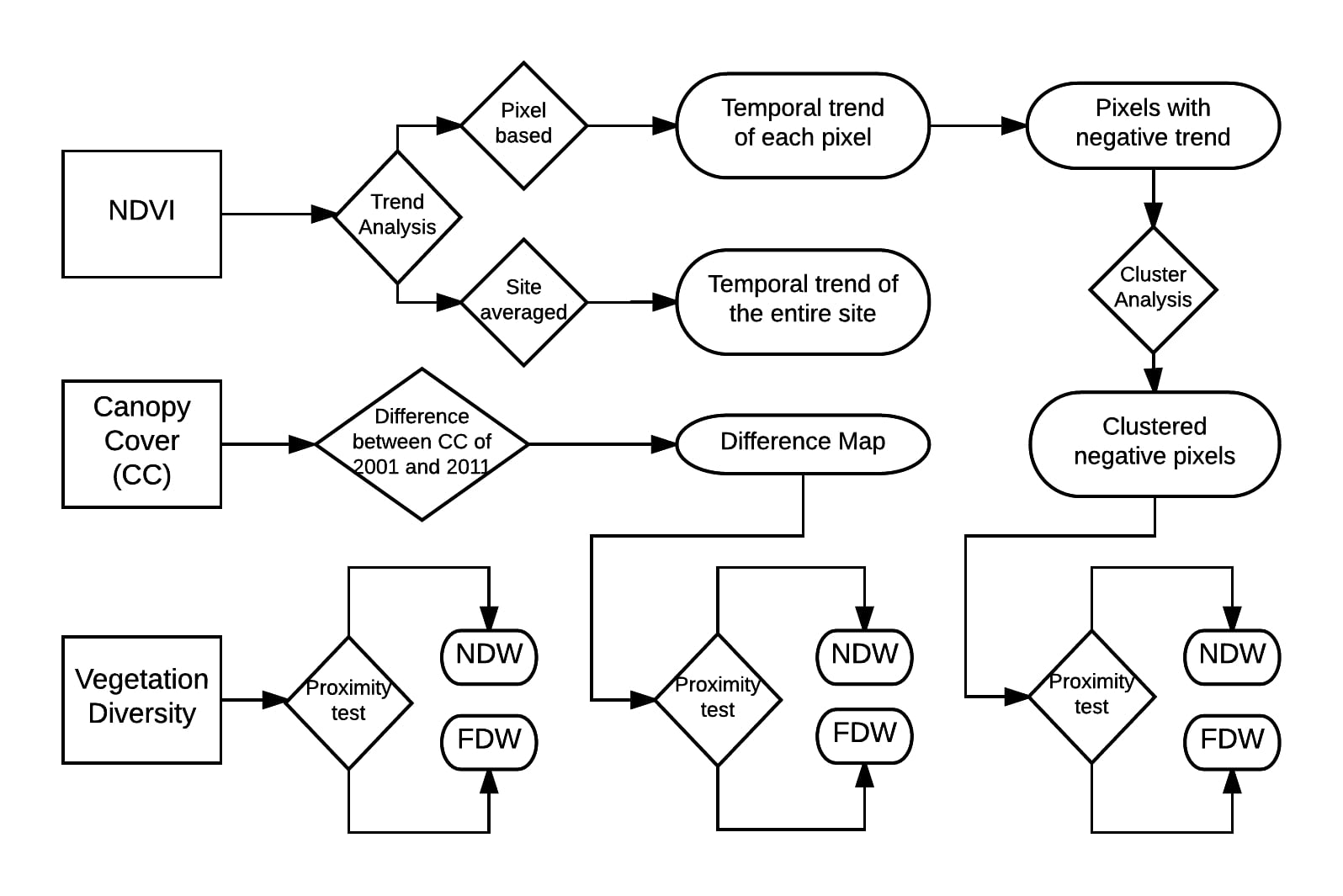


**Supplemental Figure 1:** Flowchart summarizing methods and procedures followed in this study. Three main components (NDVI, canopy cover, and vegetation diversity) are shown on the left. Diamond boxes show processes /methods, oval boxes display products/analysis results. For details about each procedure/result refer to methods section. NDW = near-downwind and FDW = far-downwind sections of riparian site.


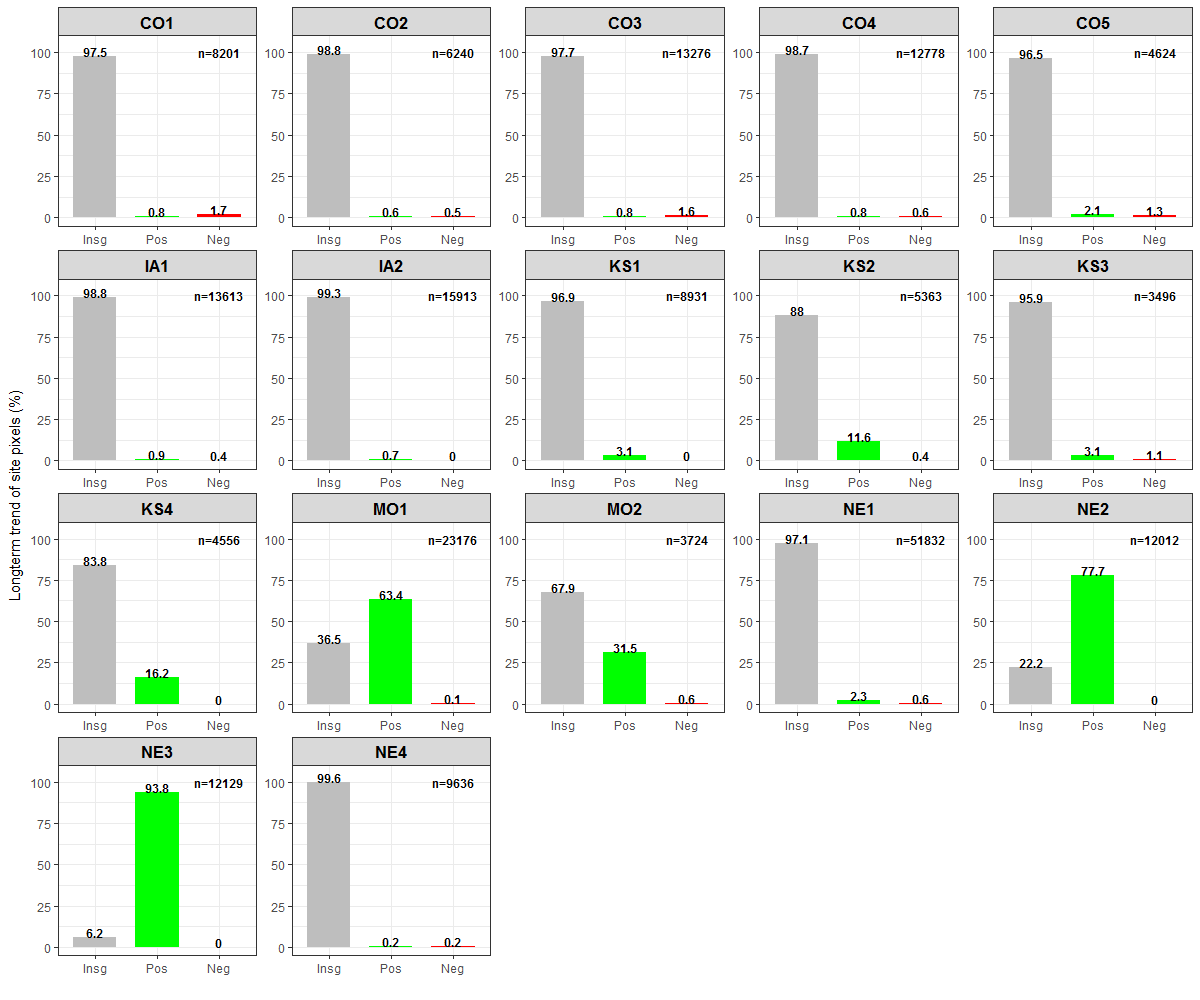


**Supplemental Figure 2:** Pixel-based trend analysis of NDVI index for spring (April and May). While “Insg” (gray) represents the proportion of pixels that exhibited no trend, “Pos” (green) and “Neg” (red) represent proportion of pixels with positive and negative trend, respectively. N represent for the total number of pixels at each site (area of each pixel is 900m2). Percentages are printed above each column. Notice that the range of y-axis can change between sites.


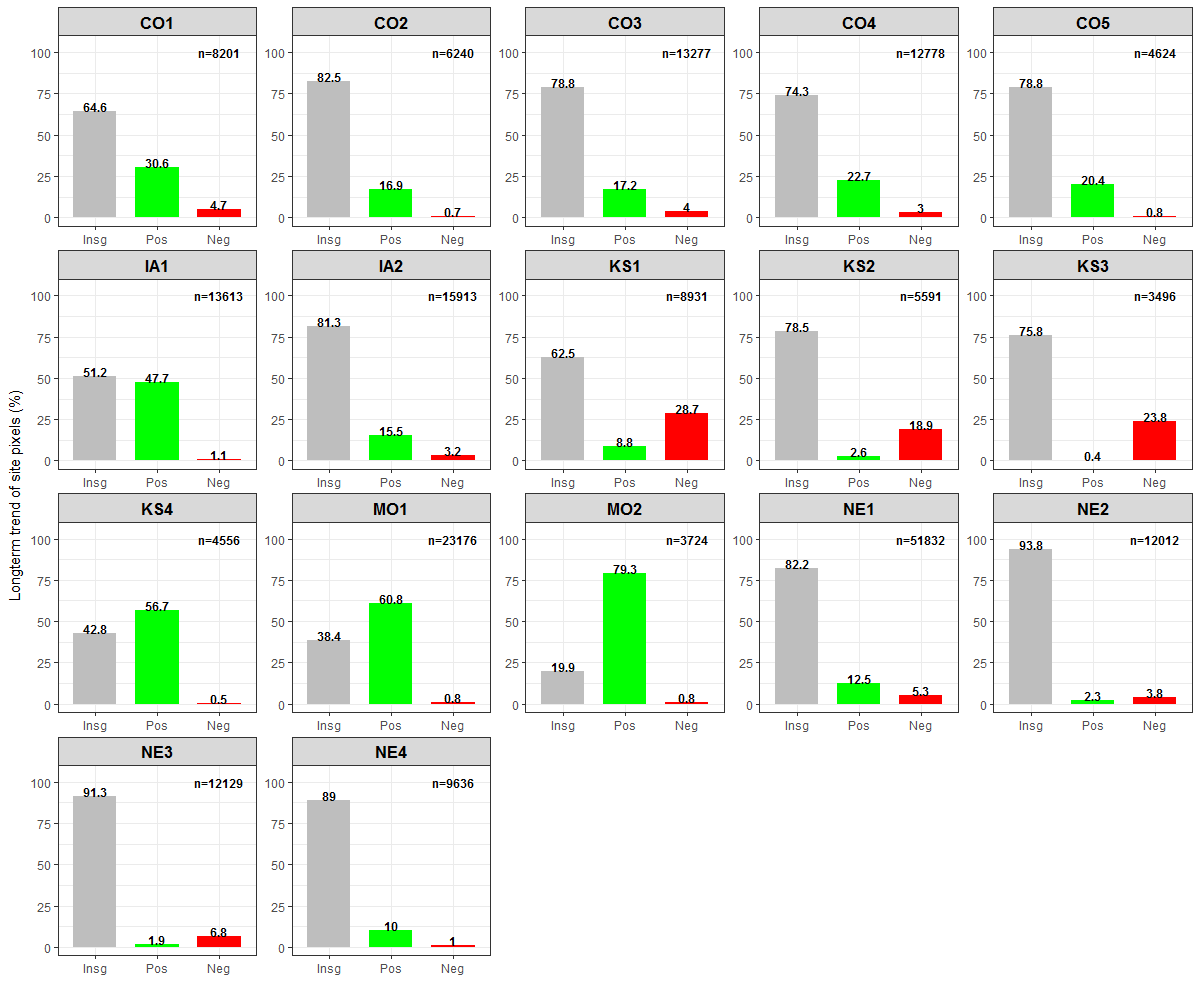


**Supplemental Figure 3:** Pixel-based trend analysis of NDVI index for summer (June and July). While “Insg” (gray) represents the proportion of pixels that exhibited no trend, “Pos” (green) and “Neg” (red) represent proportion of pixels with positive and negative trend, respectively. N represent for the total number of pixels at each site (area of each pixel is 900m^2^). Percentages are printed above each column. Notice that the range of y-axis can change between sites.


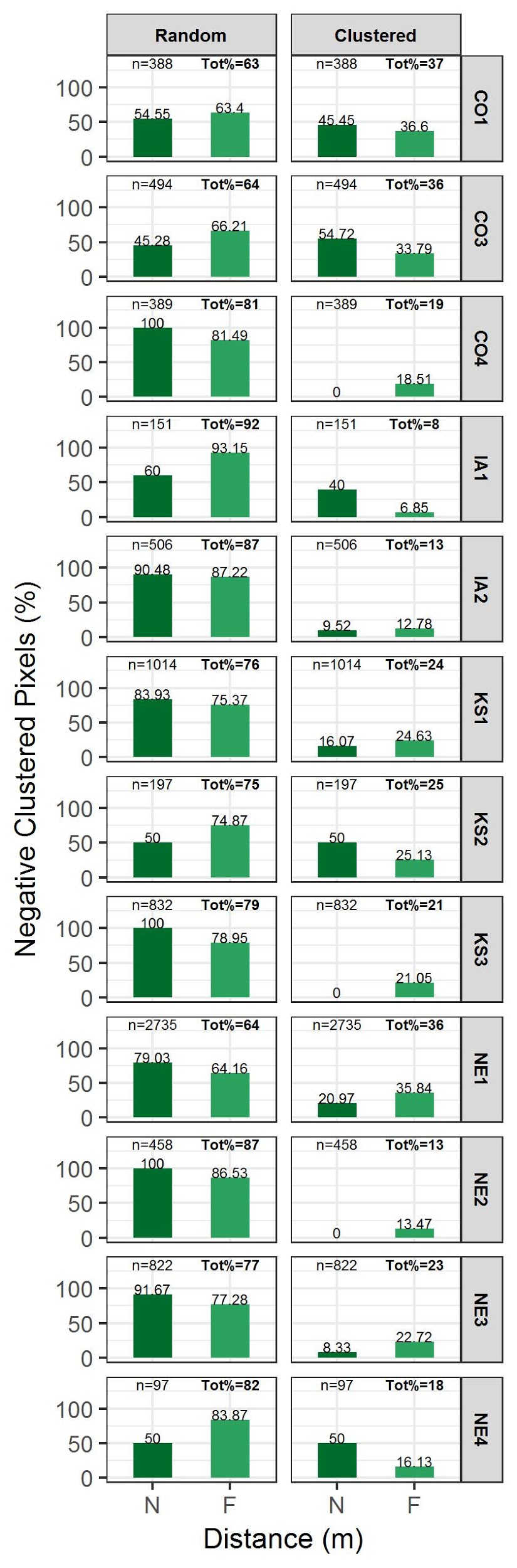


**Supplemental Figure 4:** Sensitivity analysis of cluster-analysis for summer NDVI at different selected distances to croplands. For each panel, the top titles exhibit the type of negative cluster whereas the vertical headings (right-side) display the site name. Bars show the distribution (%) of clusters in various distance (N=0-50m, F=50-400m) from the croplands. **n** represent the total number of negative pixels carried over from pixel based trend analysis. This analysis was performed for 12 sites that had greater than 1% negative pixels (CO1, CO3, CO4, KS1-3, IA1-2, and NE1-4).
